# Adaptation to Temporally Fluctuating Environments by the Evolution of Maternal Effects

**DOI:** 10.1371/journal.pbio.1002388

**Published:** 2016-02-24

**Authors:** Snigdhadip Dey, Stephen R. Proulx, Henrique Teotónio

**Affiliations:** 1 Institut de Biologie de l´École Normale Supérieure, INSERM U1024, CNRS UMR 8197, Paris, France; 2 Department of Ecology, Evolution, and Marine Biology, University of California, Santa Barbara, Santa Barbara, California, United States of America; University of New South Wales, AUSTRALIA

## Abstract

All organisms live in temporally fluctuating environments. Theory predicts that the evolution of deterministic maternal effects (i.e., anticipatory maternal effects or transgenerational phenotypic plasticity) underlies adaptation to environments that fluctuate in a predictably alternating fashion over maternal-offspring generations. In contrast, randomizing maternal effects (i.e., diversifying and conservative bet-hedging), are expected to evolve in response to unpredictably fluctuating environments. Although maternal effects are common, evidence for their adaptive significance is equivocal since they can easily evolve as a correlated response to maternal selection and may or may not increase the future fitness of offspring. Using the hermaphroditic nematode *Caenorhabditis elegans*, we here show that the experimental evolution of maternal glycogen provisioning underlies adaptation to a fluctuating normoxia–anoxia hatching environment by increasing embryo survival under anoxia. In strictly alternating environments, we found that hermaphrodites evolved the ability to increase embryo glycogen provisioning when they experienced normoxia and to decrease embryo glycogen provisioning when they experienced anoxia. At odds with existing theory, however, populations facing irregularly fluctuating normoxia–anoxia hatching environments failed to evolve randomizing maternal effects. Instead, adaptation in these populations may have occurred through the evolution of fitness effects that percolate over multiple generations, as they maintained considerably high expected growth rates during experimental evolution despite evolving reduced fecundity and reduced embryo survival under one or two generations of anoxia. We develop theoretical models that explain why adaptation to a wide range of patterns of environmental fluctuations hinges on the existence of deterministic maternal effects, and that such deterministic maternal effects are more likely to contribute to adaptation than randomizing maternal effects.

## Abstract 2

What strategies do organisms evolve to cope with an environment that varies between generations? An experimental evolution study reveals that nematode mothers evolve glycogen provisioning to protect their embryos from fluctuating oxygen levels.

## Author Summary

Mothers can influence the phenotype of their offspring, independently of the genes that they transmit to them; such phenomena are known as “maternal effects.” Theory suggests that maternal effects can be advantageous when the environment changes between generations, but a direct demonstration of this has been missing. Using the hermaphroditic nematode *Caenorhabditis elegans*, we show that the experimental evolution of maternal glycogen provisioning underlies the adaptation to a fluctuating normoxia–anoxia environment by increasing the survival of embryos under anoxic conditions. Evolution of this maternal effect only occurred in populations facing oxygen deprivation during embryogenesis every other generation (i.e., where fluctuation was regular or predictable). Unexpectedly, populations facing irregularly fluctuating oxygen levels did not evolve “bet-hedging” strategies, but instead adapted by the evolution of long-term transgenerational effects. Once evolved, maternal effects can nonetheless underlie adaptation to a wide range of temporally fluctuating environments.

## Introduction

All organisms live in temporally fluctuating environments, for example, when the environmental conditions change regularly across seasons or when environmental conditions change erratically across a range of time spans. Adaptation to these environments can happen by the evolution of different degrees of within-generation phenotypic plasticity [1–3], as long as individuals have access to reliable information early in development, allowing them to forecast the environmental conditions they will experience later in their lives [4–7]. In many cases, however, the timing of development and environmental exposure does not coincide, making it unfeasible for individuals to independently acquire and use this sort of information [8,9], as when any portion of development occurs while individuals are still in the maternal environment and, therefore, the relevant information is only available through the mother.

When environmental variation is negatively correlated between maternal and offspring generations, such as the seasonal weather changes that bivoltine insects may be faced with, selection can favor mothers that reliably cue their offspring to alter development and/or provision essential resources for offspring survival [5,10,11]. These maternal effects have been termed “anticipatory” maternal effects or transgenerational phenotypic plasticity [12–14]. Similarly, positively correlated environments, such as that of relatively constant and slowly fluctuating environments, can lead to the evolution of anticipatory maternal effects [5,15] and of a phenotypic “memory” of past environments [7,14,16–18]. In general, the rate of change in trait mean of a population because of selection equals its covariance with relative fitness [19,20]. In fluctuating environments, selection creates a positive covariance between the alleles individuals pass on to their progeny and the environment their mothers have experienced [21–23]. But because maternal effects may involve transfer of resources to offspring, within-generation developmental and physiological trade-offs can determine whether or not maternal effects will evolve [11].

When environmental variation is uncorrelated between maternal-offspring generations, mothers cannot reliably provision or cue their offspring. In these environments, however, “bet-hedging” or “risk-spreading” maternal effects may be favored, since mothers who bear young with a randomized mix of phenotypes can ensure that at least some will be able to survive in order to reproduce [24–28]. Randomized offspring phenotypes could arise as well through developmental instability and be selectively favored, and these would be largely indistinguishable from maternal effects [18,29,30]. However, mothers can be implicated whenever differential provisioning of resources is required to produce alternative offspring phenotypes. In a similar vein, maternal strategies producing a generalist phenotype may be selected in fluctuating environments. Such “conservative” bet-hedging is also difficult to identify because it does not result in environment-specific fitness effects or increases in the variance of the relevant traits [30,31]. In each of these cases, however, the bet-hedging strategy will spread in a population because it increases the long-term population growth rate by decreasing the among-generation variance in fitness, even if at the expense of short-term survival and reproduction [32–36].

Maternal effects are common in a wide variety of plants and animals [12,30,37–44], and adaptive maternal effects are usually defined as those that increase offspring fitness [12,45]. Here, we take the strict definition that for maternal effects to be adaptive, they must evolve because of selection on offspring and result in increased long-term population growth rates. Otherwise, maternally mediated trait changes in the offspring could emerge as a result of maternal selection [46]. For instance, maternal effects are widespread in placental animals, but they are likely to have evolved because of a shift from selection in pre- to post-copulatory traits enhancing the mothers’ performance when multiple mates are available [47]. One further criterion for the existence of adaptive maternal effects is that they can evolve at the expense of compromised maternal performance [11,48].

Experimental studies explicitly testing for the adaptive nature of maternal effects in temporally fluctuating environments are rare, and the results from these studies are equivocal ([46,49]; see Discussion). Using experimental evolution in the nematode *Caenorhabiditis elegans*, we designed regimes with different degrees of maternal-offspring environmental correlation that can in principle select for maternal effects and then test the evolved populations for adaptive maternal effects. We used a previously defined stress that has been studied at the developmental and physiological levels and that has the potential to reveal the adaptive nature of a maternal effect. In particular, when hermaphrodites of *C*. *elegans* experience a hyperosmotic stress, the embryos they lay show reduced hatchability to the first larval stage when exposed to anoxia [50]. This is due to a metabolic tradeoff between the hermaphrodites’ ability to produce glycerol, an osmolyte and lipid membrane precursor, during growth from larval stages to maturity and the ability of hermaphrodites to provision their broods with glycogen, an essential glucose energetic store for embryogenesis and larval hatching in anoxia [50–52]. Taking advantage of this hermaphrodite-embryo constraint between survival in hyperosmotic and anoxic environments, we specifically ask if adaptation to a novel fluctuating normoxia–anoxia environment can be achieved by the evolution of maternal strategic glycogen provisioning.

We performed sixty generations of *C*. *elegans* experimental evolution in three environmental regimes that varied in the degree of mother–offspring normoxia–anoxia correlation. Anticipatory maternal effects were expected to evolve in populations facing a negatively correlated normoxia–anoxia regime across mother–offspring generations, with hermaphrodites experiencing a normoxia hatching environment being able to prepare their progeny for anoxia by increasing glycogen provisioning. In turn, bet-hedging could be favored in populations experiencing an uncorrelated normoxia–anoxia regime across mother–offspring generations because hermaphrodites raised in either normoxia or anoxia who randomized glycogen provisioning could reduce the population fitness variance between generations. Adaptation to a positively correlated regime of consecutive anoxia generations was expected to occur mostly through the evolution of developmental or physiological mechanisms expressed constitutively by embryos leading to their higher survival under anoxia. Finally, we investigate whether the observed level of adaptation was consistent with the expected population genetics of maternal effects under our experimental evolution design.

Hereafter, we will refer to bet-hedging or risk-spreading maternal effects as “randomizing” maternal effects. Conversely, we refer to “deterministic” maternal effects when the mother’s phenotype in a given environment has a consistent effect on the offspring phenotype. We define maternal effects in this way to minimize confusion between the developmental and physiological mechanisms of maternal resource provisioning (or offspring cueing) with the evolutionary outcome of selection and the principle of long-term fitness maximization that applies to any population faced with temporally fluctuating environments. A formal justification for the use of this terminology can be found in [11].

## Results

### Fitness under Anoxia in a High-Salt Normoxia Adapted Population

We first evolved a population, previously adapted for 140 generations to the life cycle and density conditions to be employed during experimental evolution [53,54], for 50 generations in an environment where post-hatching larvae-to-adult rearing took place at 305 mM NaCl [55]. This was done so that adaptation to the novel fluctuating oxygen levels during embryogenesis and larval hatching would not be confounded with adaptation to a hyperosmotic challenge throughout the several life stages. A single salt-adapted population was then used as the ancestor for all experimental populations employed in the present study.

**Fig 1:**
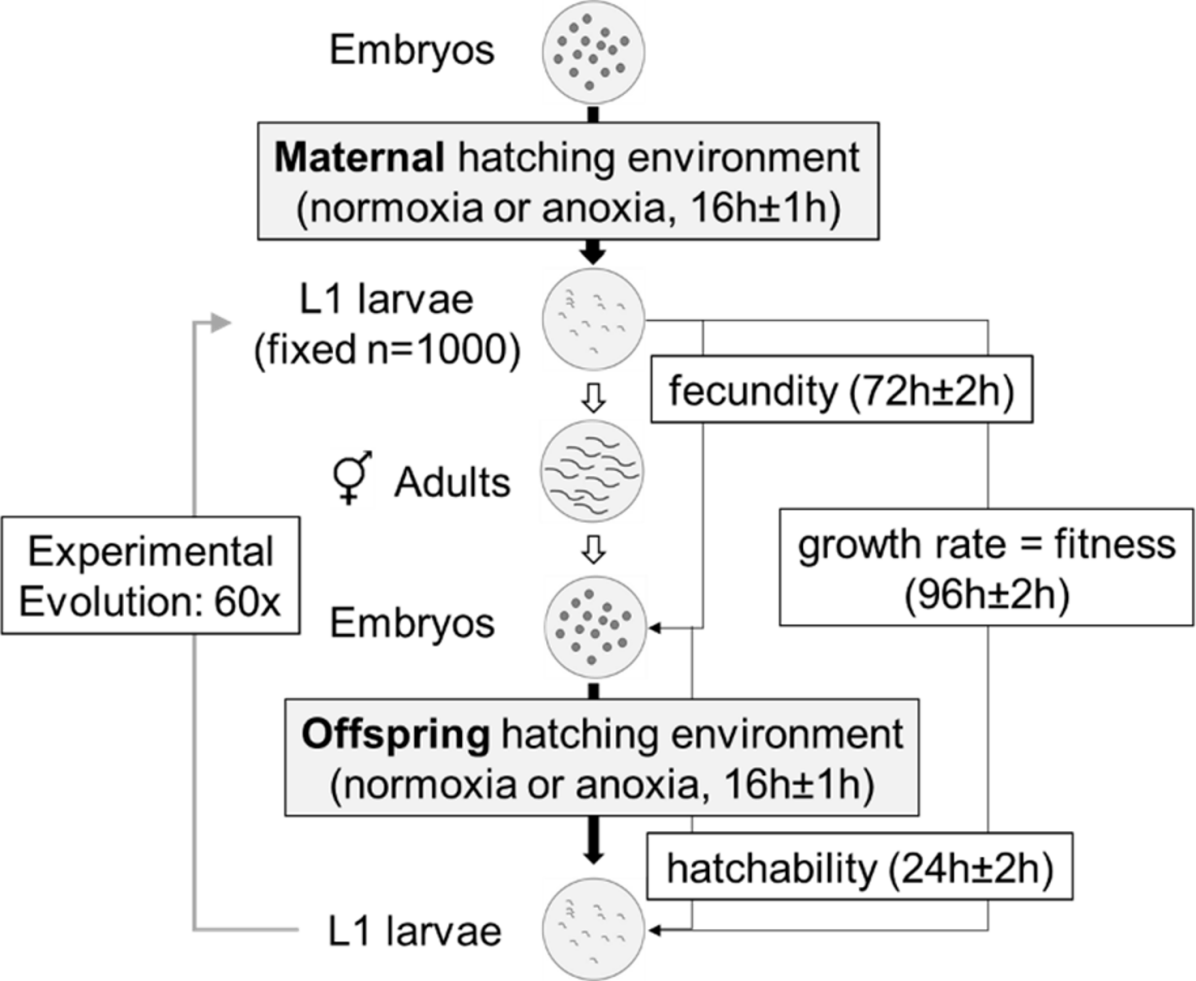
*C*. *elegans* laboratory life cycle in normoxia-anoxia environments. Schematic of the 4-day life cycle under high salt conditions from the first larval stage (L1) to adulthood, and varied oxygen levels from embryo to L1 hatching over two generations. Generations were non-overlapping, with densities being kept constant from L1 to adulthood at 10^3^ per Petri dish plate [54,55]. This life cycle was employed during experimental evolution to the novel fluctuating normoxia–anoxia environment and to characterize experimentally evolved populations in terms of growth rates (fitness), fecundity, hatchability, oocyte size, and glycogen content (see below).

**Fig 2:**
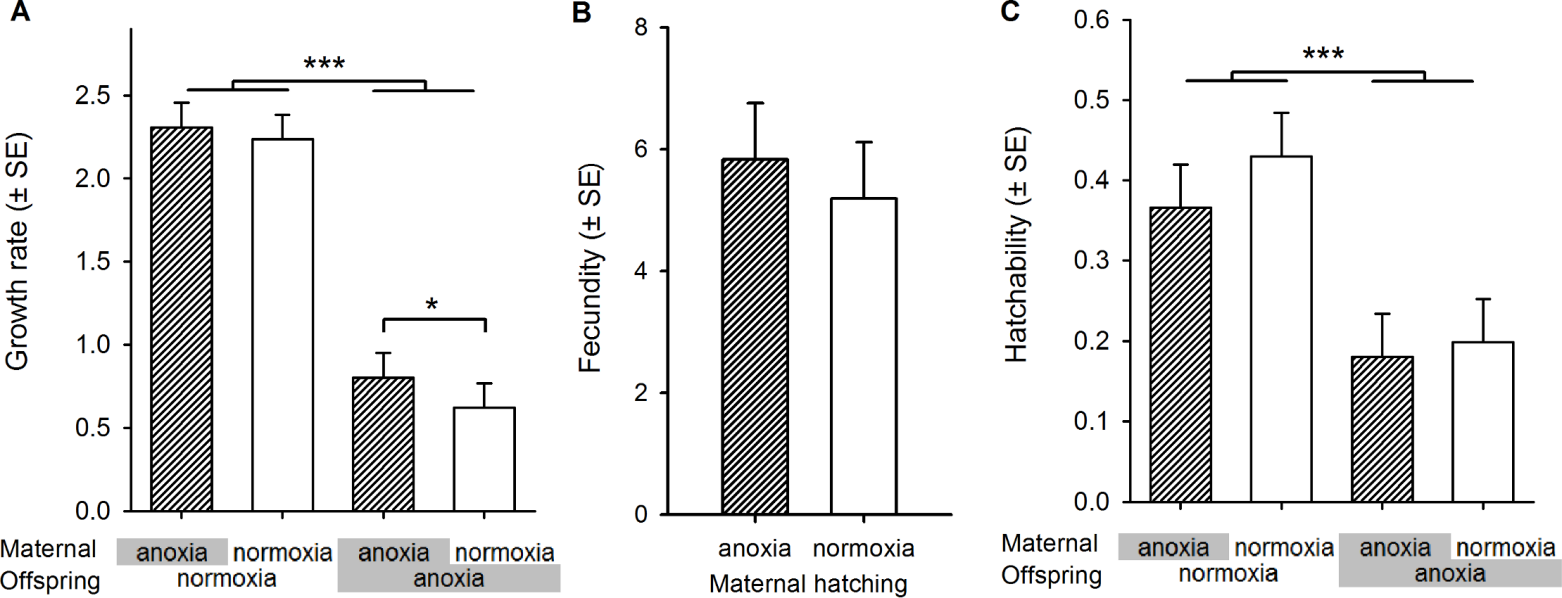
Fitness and fitness components in the ancestor high-salt normoxia-adapted population. (A) The natural logarithm of the growth rate of the high-salt-adapted population (ancestral to the populations used in the present study) across the four possible maternal–offspring hatching environments. Over one full life cycle, ln growth rate is an estimate of the absolute fitness with positive values indicating that the population would grow exponentially with unlimited resources. (B) The fecundity of hermaphrodites deprived of oxygen during embryogenesis is not affected relative to normoxia rearing condition. (C) Embryo hatchability to the L1 stage after anoxia exposure is severely hampered, independently of maternal hatching environment. (A–C) We employed linear mixed-effect models (LMM) [56], taking sample thawing block as a random factor and maternal hatching treatment, in the case of fecundity, or maternal and offspring hatching treatments, in the case of growth rate and hatchability, as fixed factors (see Materials and Methods for further details). Planned contrasts were then done with post-hoc Tukey *t* tests [57] and LMM-corrected Kenward-Roger (KR) degrees of freedom [58]: * *p* < 0.05, *** *p* < 0.001. Data deposited in the Dryad repository: http://dx.doi.org/10.5061/dryad.56bb4 [59].

We then found that over a two-generation assay in larvae-to-adult high-salt conditions (Fig 1; see Materials and Methods), the high-salt-adapted population showed a marked growth rate reduction when embryos were exposed to anoxia (Fig 2A; t_148_ = 30.4, *p* < 0.001; see Materials and Methods for details on statistical modeling). Reduction in growth rate was more evident when the maternal generation experienced a normoxia hatching environment (t_149.8_ = 2.4; *p* < 0.02). As our assay covered a full life cycle in the environmental conditions to which the high-salt population had adapted, except for oxygen deprivation, these results show that anoxia exposure reduces the absolute population fitness.

Differences among maternal–offspring oxygen treatments in absolute fitness could be due to lower fecundity of hermaphrodite mothers after experiencing anoxia while they were embryos and/or due to lower embryo survival to first-stage larvae when faced with anoxia in the following generation (i.e., hatchability; Fig 1). We found that hermaphrodites exposed to anoxia when embryos have similar fecundity as those exposed to normoxia (Fig 2B; t_39_ = 1.4; *p* = 0.26). In contrast, the hatchability was halved when embryos were exposed to anoxia relative to when embryos were exposed to normoxia (Fig 2C; t_44.5_ = 4.9; *p* < 0.001), independently of whether or not their mothers experienced oxygen deprivation as embryos (t_44.5_ = 0.59; *p* = 0.56). There was thus ample opportunity for selection on embryo hatchability under anoxia, and presumably less opportunity for selection on adult fecundity. Furthermore, there is little evidence that the ancestral population expressed a maternal effect, since embryo hatchability under anoxia did not depend on the maternal environment. This means that, in the ancestral population, mothers likely did not prepare their progeny for future anoxia exposure if they themselves experienced oxygen deprivation during embryogenesis and hatching.

### Opportunity for Adaptation during Experimental Evolution

Characterization of 405 single-nucleotide polymorphisms (SNPs) across the genome showed that the ancestor population had appreciable standing genetic variation from which adaptation to the novel fluctuating oxygen level environments could occur (S1 Fig). This population was nevertheless highly inbred, because most reproduction occurred by self-fertilization [60], as hermaphrodites cannot outcross each other [61] and males were at low frequencies (from [55], males were initially at around 5%, and during the experimental evolution males were never observed). The fact that males were at low frequencies ensured little expression of a sexual conflict between males and hermaphrodites that could have otherwise limited selection for maternal effects [62]. In addition, the fact that self-fertilization was the predominant breeding mode diminished the chance for the expression of parent–offspring conflicts (cf., [63]) because, barring overdominant loci (*C*. *elegans* being diploid) [53] and mutational input [64], hermaphrodites are genetically identical to all of their progeny.

**Fig 3:**
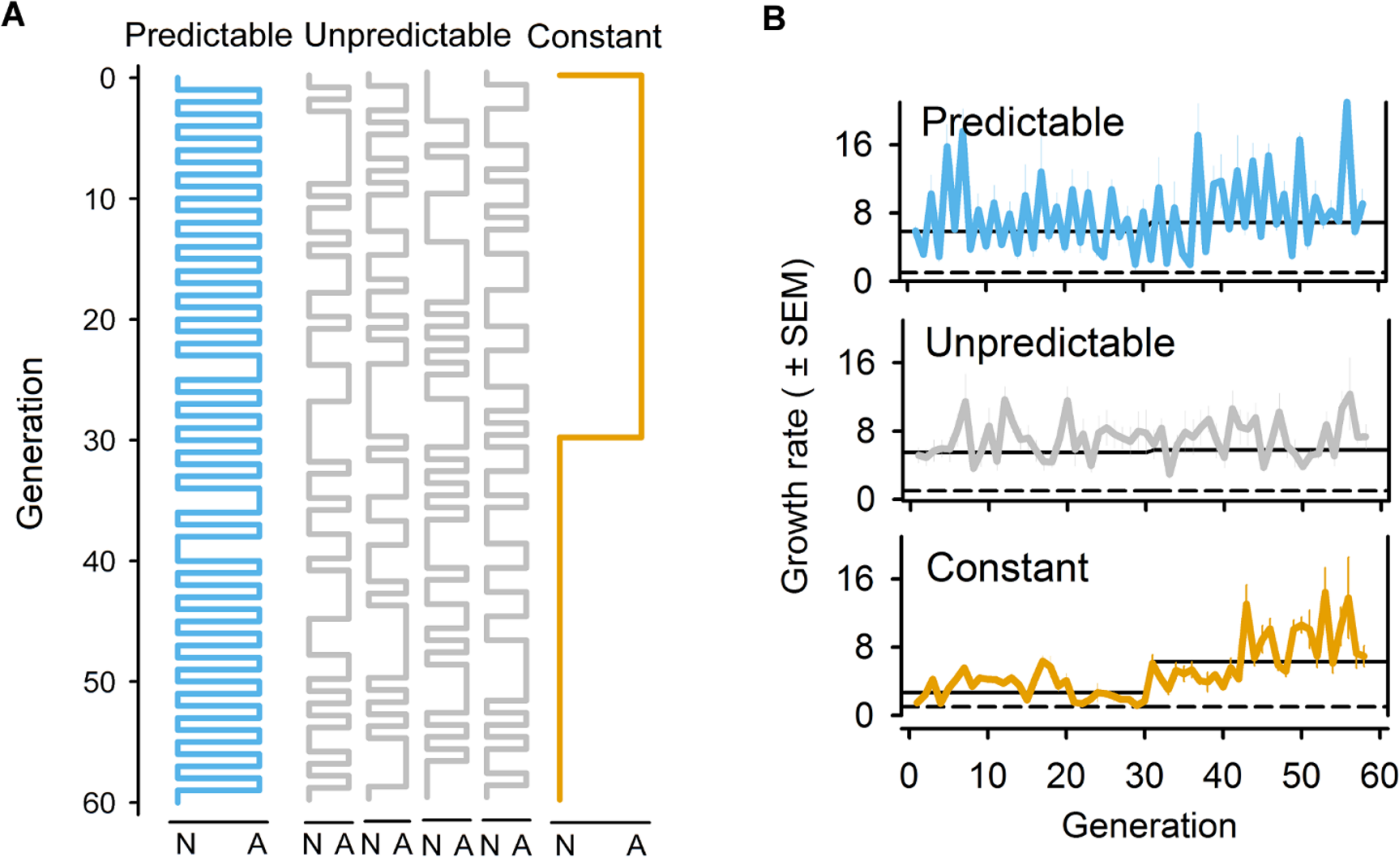
Experimental evolution under fluctuating normoxia–anoxia environments. (A) Schematic of the fluctuating sequences of normoxia (N) and anoxia (A) environments employed during the 60 generations of experimental evolution. See S2 Fig for the autocorrelation and spectral decomposition of environmental fluctuations at several intervals [65,66]. (B) Mean expected growth rates during experimental evolution, measured from generation 1 to generation 58. Error bars show one standard error of the mean among replicate populations. Solid lines show the geometric mean growth rate for the first 30 generations or the last 30 generations of experimental evolution. Dashed lines show the growth rate below which the population would go extinct, under the fixed density conditions of the life cycle. Environmental sequences and data deposited in the Dryad repository: http://dx.doi.org/10.5061/dryad.56bb4 [59].

Experimental evolution to fluctuating normoxia–anoxia environments was done for 60 generations under three different regimes (Figs 3A and S2). The “predictable” regime imposed alternating normoxia and anoxia conditions every other generation. In this regime, the probability of mothers and progeny sharing the same environment was 0.05 across the 59 mother–offspring transitions. There were four replicate populations undergoing independent experimental evolution in the predictable regime. In the “unpredictable” regime, the probability of mothers and progeny sharing the same environment was 0.46 across the 59 mother–offspring transitions. We imposed four different unpredictable environmental sequences, each 2-fold replicated, in order to encompass a range of normoxia–anoxia fluctuations across multiple generations (S2 Fig; cf., [65,66]). Finally, a “constant” regime was characterized by 30 consecutive generations of anoxia followed by 30 consecutive generations of normoxia, this regime being 4-fold replicated. In all regimes, the frequency of normoxia and anoxia generations was 0.5 across the 60 generations. As was the case during previous laboratory adaptation [54,55], all 16 experimental populations were cultured under nonoverlapping 4-day life cycles at fixed N = 10^4^ densities and 305 mM NaCl from the larval L1 stage to adulthood (Fig 1; see Materials and Methods). We expected effective population sizes to be on the order of N_e_ = 10^3^ during experimental evolution [53,67].

During experimental evolution, we subsampled the suspended embryo solution after reproduction in order to determine the density of L1 hatched larvae competing to be part of the next generation (see Materials and Methods). This assay thus gave us the expected growth rates of each population if we were to relax the fixed density dependence from L1 to adulthood (Fig 1). Expected growth rates during experimental evolution were similar among predictable and unpredictable populations, and always high enough to maintain the required population size for continued culturing (Fig 3B). In contrast, the expected growth rates of the constant populations during the first 30 generations were lower relative to the other regimes, though still always above replacement rates. These expected growth rate dynamics during experimental evolution suggest that the extent of genetic drift due to finite population sizes and predominant self-fertilization may have been greater in the constant populations than under the other regimes. If so, then the effective selection on genotypes expressing embryo-specific developmental or physiological mechanisms enhancing embryo survival under anoxia would not be comparable among regimes. This does not seem to have been the case, however, since genome-wide SNP characterization of evolved populations indicated a great loss of genetic variation during experimental evolution, but with populations from all regimes showing similar standing levels after 60 generations (S3 Fig).

### Adaptation to Normoxia–Anoxia Environments

**Fig 4:**
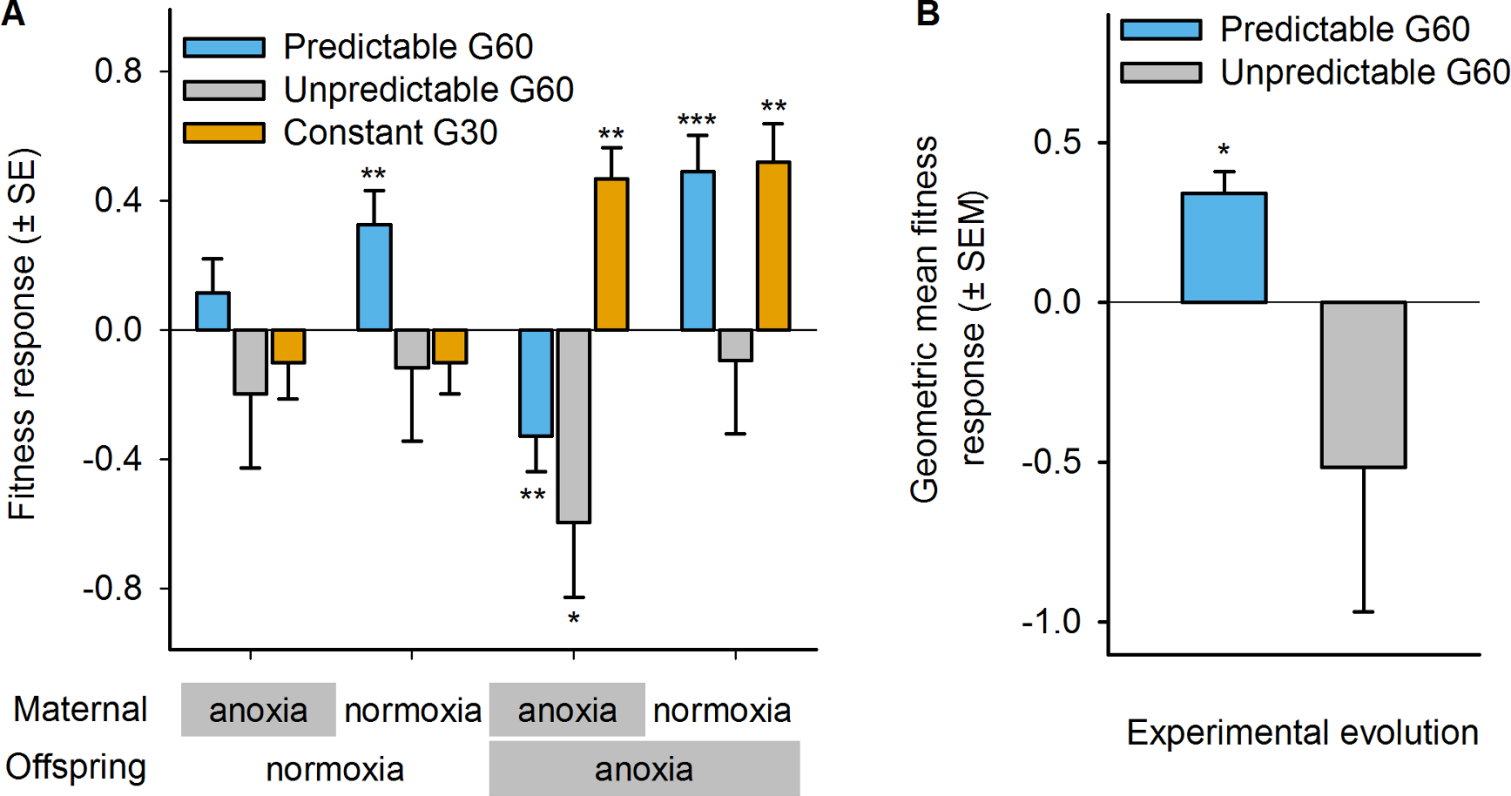
Adaptation to normoxia–anoxia environments. (A) Relative fitness of predictable (blue), unpredictable (grey), and constant (orange) populations to the ancestor population (zero line), across the four combinations of maternal–offspring environments. Ancestor and evolved populations were concurrently assayed after two generations of “common garden” maintenance to account for thawing assay block effects (see Materials and Methods). Relative fitness is calculated as the natural logarithm of evolved growth rates over the mean ancestral growth rate per block. Mean and error least square estimates are shown after separate LMM for each experimental regime, taking replicate population as a random factor and the maternal–offspring hatching treatment as a fixed factor. Significant fitness responses relative to the ancestor population (done with post-hoc Student *t* tests, with LMM-corrected KR degrees of freedom) are shown above each bar: * *p* < 0.05; ** *p* < 0.01; *** *p* < 0.001. (B) From (A), the geometric mean fitness is calculated over anoxia–normoxia and normoxia–anoxia absolute fitness separately for each of the four replicate predictable populations, or over all mother–offspring environments for each of eight unpredictable populations, and compared to the absolute fitness of the ancestral population. Error bars show one standard error of the mean among replicate populations. Significance of a Student *t* test is shown above the bar: * *p* < 0.05. Data deposited in the Dryad repository: http://dx.doi.org/10.5061/dryad.56bb4 [59].

After 60 generations of experimental evolution, the predictable populations showed an increase in fitness relative to the ancestral population when hermaphrodites experienced normoxia and their embryos experienced anoxia (Fig 4A; Student t_21.3_ *p* = 0.002), but showed no change in relative fitness when mothers experienced anoxia and their progeny experienced normoxia (t_17.7_ *p* = 0.3). Note that all fitness responses are measured as the natural logarithm of evolved growth rates over ancestral growth rates, being thus tested for significant differences to zero, the ancestral adaptive state (see Materials and Methods). There were also changes in relative fitness in the two mother–offspring environments that were largely absent from the sequences imposed on the predictable populations during experimental evolution, which, therefore, could only have evolved as a correlated response. In particular, under two successive generations of normoxia, relative fitness increased (t_17.7_ *p* = 0.007), while under two successive generations of anoxia, relative fitness decreased (t_18.6_ *p* = 0.007).

In contrast to the predictable populations, the unpredictable populations did not improve their relative fitness in any combination of mother–offspring environments after experimental evolution (Fig 4A). However, and similarly to the predictable populations, the unpredictable populations showed a significant relative fitness reduction when exposed to anoxia for two consecutive generations (t_8.8_ *p* = 0.02).

As expected, the constant populations evolved increased relative fitness in anoxia after 30 generations of continuous exposure to anoxia (Fig 4A). The fitness response was not dependent on the maternal environment (maternal anoxia: t_5.4_ *p* = 0.005; maternal normoxia: t_9.9_ *p* = 0.009).

In temporally fluctuating environments, the appropriate measure of adaptation is the geometric mean of environment-specific fitness. The presence of maternal effects does not change this principle but does require averaging over the frequencies of all the pairwise environmental transitions experienced by a population during its history. When doing these calculations, we can show that predictable populations adapted to the fluctuating environment they experienced during their history (Fig 4B; t_3_ *p* = 0.01, see Materials and Methods). In the unpredictable populations, there was a trend toward a decrease in geometric mean fitness, though it was not significant due to great replicate population heterogeneity (t_7_ *p* = 0.29). As shown in Fig 4A, constant populations adapted to their anoxia environment.

Although populations from all the regimes were exposed to the same number of anoxia generations across the 60 generations of experimental evolution, a possible explanation for the lack of an adaptive response in the unpredictable populations could be that, unlike the predictable populations, they faced irregular short stretches of consecutive anoxia generations, which could have eroded genetic variation more effectively. We ruled out that lack of genetic variation limited the scope for adaptation in the unpredictable populations, since they did not have abnormally low genetic variation when compared to populations from the other regimes (S3 Fig). Moreover, we found that the constant populations, despite relatively small population sizes and great loss of genetic variation during the first 30 generations under anoxia, adapted to normoxia when subsequently challenged for another 30 generations under normoxia (S4 Fig).

### Evolution of Maternal Effects

**Fig 5:**
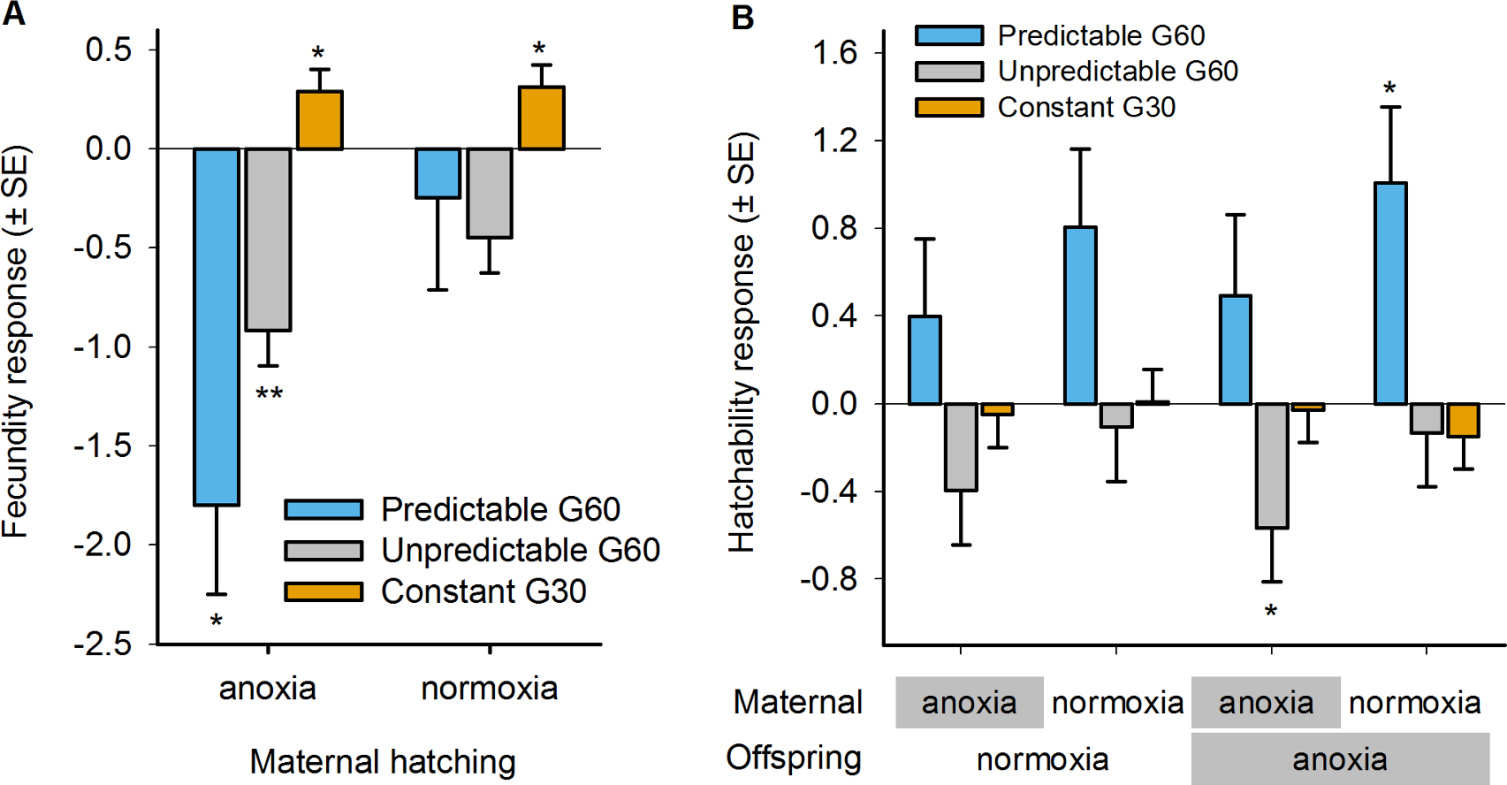
Evolution of maternal effects. Fecundity (A) and hatchability (B) in the predictable (blue) and unpredictable (grey) populations at generation 60, and in the constant populations at generation 30 (orange), relative to the ancestral population (zero line). Note that, as for fitness, evolutionary responses are measured as the natural logarithm of evolved trait values over the ancestral mean trait value per block (see Materials and Methods). Mean and error least square estimates are shown after separate LMM for each experimental regime, taking replicate population as a random factor and maternal hatching (A) or maternal–offspring hatching treatment (B) as fixed factors. Significant evolutionary responses (post-hoc Student *t* tests, with LMM-corrected KR degrees of freedom) are shown above each bar: * *p* < 0.05; ** *p* < 0.01. Data deposited in the Dryad repository: http://dx.doi.org/10.5061/dryad.56bb4 [59].

One signature for the evolution of adaptive deterministic maternal effects is that population fitness depends on the combination of maternal and offspring environment in a nonadditive fashion, as was the case in the predictable populations. However, direct evidence for the evolution of an adaptive deterministic maternal effect is that embryo hatchability to the first larval stage must have evolved to depend on both the maternal and offspring environments. This was indeed the case under the predictable environmental regime. Although we found that hermaphrodites who experienced anoxia early in life had lower fecundity as compared with the ancestral hermaphrodites (Fig 5A; t_4,1_ *p* = 0.02), hatchability under anoxia increased significantly only when the maternal generation experienced normoxia (Fig 5B; t_5_ *p* = 0.03). There was, nonetheless, an overall tendency toward higher hatchability, with a marginally significant increase in hatchability when hermaphrodites and embryos were exposed to normoxia (t_4,5_ *p* = 0.07). Since there was no decrease in relative fitness under the anoxia–normoxia transition (see Fig 4A), increased hatchability must have compensated for reduced fecundity. These patterns clearly indicate the evolution of an adaptive deterministic maternal effect: predictable hermaphrodites exposed to normoxia as embryos produce embryos with the altered phenotype of high anoxia hatchability.

Under the unpredictable regime, there is no prospect for selection to favor a deterministic maternal effect, yet there is opportunity to respond to selection by increasing the geometric mean of fitness over the four types of environmental transition the populations experienced. In terms of two-generation fitness components (Fig 1), we observed a general decrease in fecundity, the more so when the hermaphrodites experienced anoxia early in life (Fig 5A, anoxia: t_4.9_ *p* = 0.004; normoxia: t_4.9_ *p* = 0.06). Hatchability did show a maternal effect in that under anoxia–anoxia but not under normoxia–anoxia it significantly decreased relative to the ancestral population (Fig 5B, t_9.4_ *p* = 0.05). But while there is evidence of a maternal effect on hatchability, there is no evidence that it was adaptive, since the unpredictable populations did not show an increase in geometric mean fitness (Fig 4B).

In the constant populations, adaptation could have proceeded through general developmental or physiological mechanisms that increased performance in anoxia, even if they decreased performance under other environmental sequences. We found that adaptation proceeded through an increase in hermaphrodite fecundity that was environment-independent (Fig 5A; anoxia: t_4.5_ *p* = 0.05; normoxia: t_4.5_ *p* = 0.04) without any decrease in hatchability (Fig 5B).

### Evolution of Maternal Glycogen Provisioning

**Fig 6:**
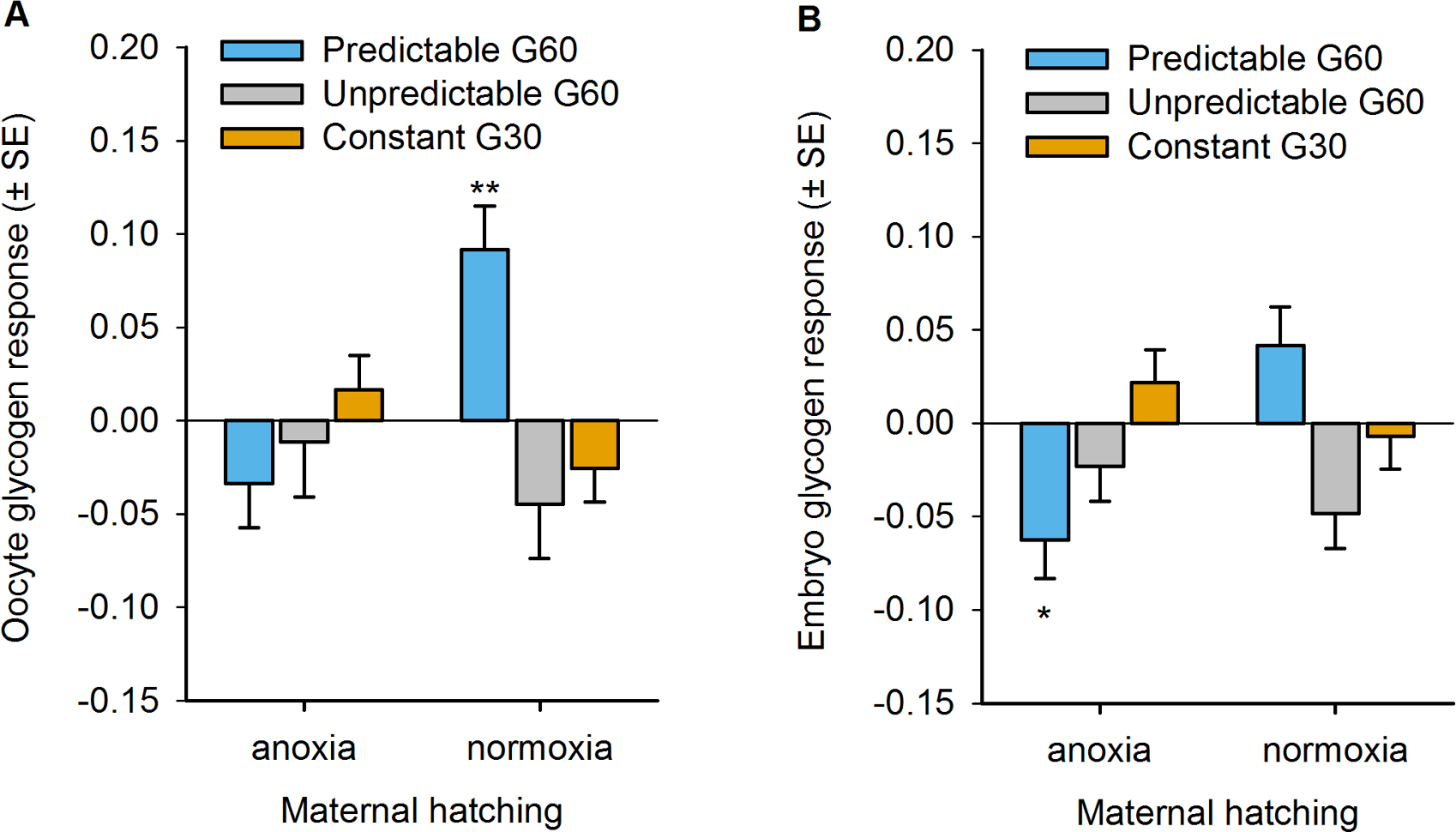
Evolution of maternal glycogen provisioning. Glycogen content is quantified in iodine-stained hermaphrodites at the time of reproduction during experimental evolution. Oocyte (A) and in utero embryo (B) glycogen content of the predictable (blue) and unpredictable (grey) populations at generation 60, or constant populations at generation 30 (orange), relative to the ancestral population (zero line). See S5 and S6 Figs and Materials and Methods for assay details and controls. Mean and error least square estimates are shown after separate LMM for each experimental regime, taking replicate population and individual hermaphrodite as random factors and maternal treatment as a fixed factor. Significant evolutionary responses (Student *t* tests, with LMM-corrected KR degrees of freedom) are shown above each bar: * *p* < 0.05; ** *p* < 0.01. Data deposited in the Dryad repository: http://dx.doi.org/10.5061/dryad.56bb4 [59].

We confirmed the hypothesis that the maternal effect that evolved in the predictable populations was due to increased hermaphrodite glycogen provisioning of their embryos [50]. We quantified glycogen content in the oocytes and in utero embryos in hermaphrodites at the usual time of reproduction during experimental evolution, i.e., before the oxygen level treatment that their embryos would have been exposed to (Fig 1; see Materials and Methods). After 60 generations under the predictable regime, we see a clear pattern of increased glycogen content when hermaphrodites experience normoxia (Fig 6). At the oocyte stage, this manifests as a significant increase in glycogen content under maternal normoxia relative to the ancestor (t_10.57_ *p* = 0.003), while at the embryo stage we observe a significant decrease in glycogen content under maternal anoxia relative to the ancestor (t_6.2_ *p* = 0.02). Taken together, these findings show that, for the predictable populations, hermaphrodites increased glycogen provisioning under conditions where their offspring were likely to experience anoxia. See S5 Fig for values in the ancestral population, and S6 Fig for unstained controls.

For completeness, we also measured the oocyte and embryo glycogen content in hermaphrodites from the other two regimes even though there was no expectation of an evolutionary change, given the fitness and fitness component results (Figs 4 and 5). Generation 60 unpredictable hermaphrodites reared in normoxia showed a marginal reduction in embryo glycogen content (t_4.4_ *p* = 0.06), but otherwise, neither unpredictable hermaphrodites nor constant hermaphrodites at generation 30 showed much changes in glycogen content (Fig 6).

There could have been evolution of oocyte size improving glycogen utilization (through increased glycogen concentration, for example) and/or diminished oxidative stress (through decreased surface-area tension and gas exchange, for example). We found, however, little evidence for evolution of oocyte size (S7 Fig). We further tested whether increased variation among oocyte and embryo glycogen content within hermaphrodites evolved in the unpredictable populations, as expected with the evolution of a randomizing maternal effect or increased developmental instability. We failed to detect any such increases in within-brood trait variation, as there was, if anything, a decrease with evolution (S8 Fig).

### The Population Genetics of Maternal Effects during Experimental Evolution

To understand how the particular sequence of environmental transitions across generations determined the course of experimental evolution (Figs 2A and S2), we developed mathematical models to calculate the probability that an allele conferring maternal effects would invade the ancestral population (see Materials and Methods) [35,68]. We considered a scenario with two offspring phenotypes, one that had higher fitness under anoxia and the other that had higher fitness under normoxia. Deterministic maternal effects were defined by an allele that always produced offspring with the phenotype suited to the environment that the mother did not experience. The randomizing maternal effects allele produced a brood with a fixed fraction of each phenotype, with the fraction chosen in order to maximize the population geometric mean fitness.

**Fig 7:**
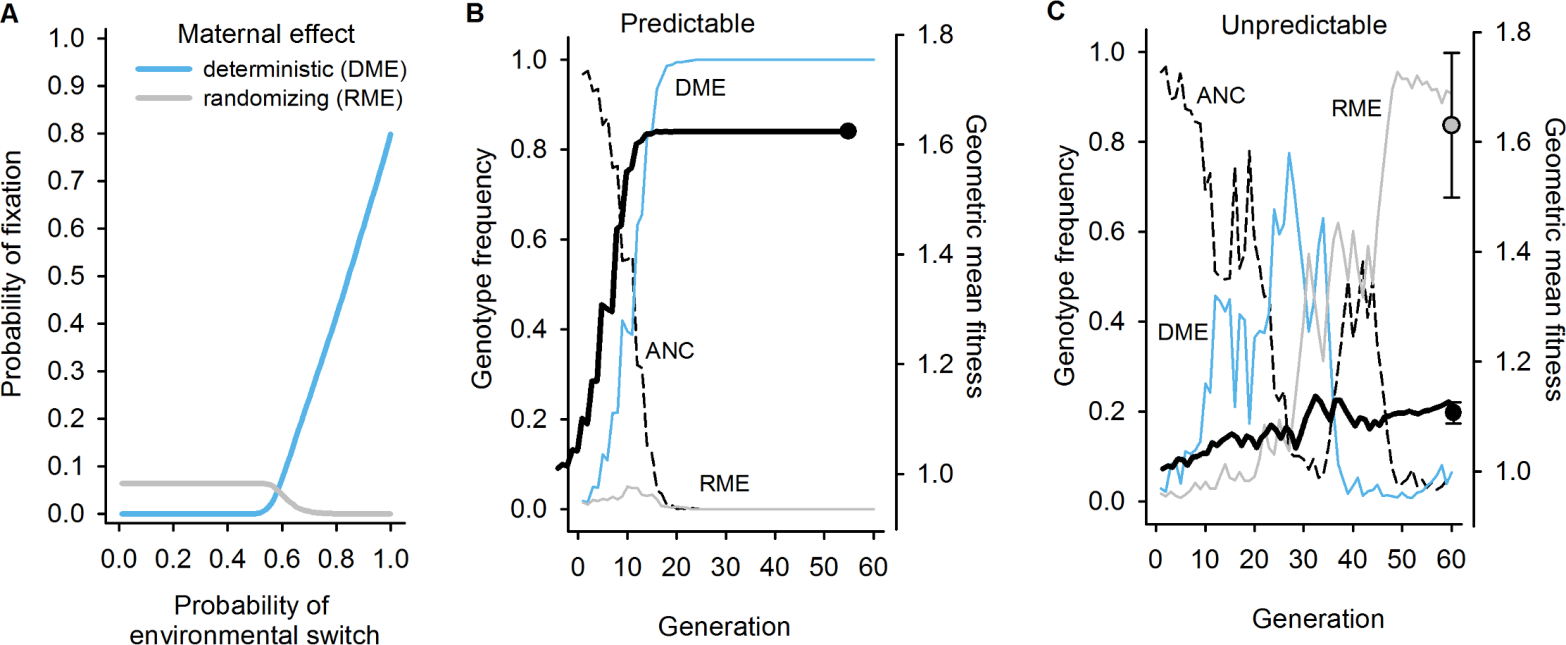
Expected population genetics of maternal effects during experimental evolution. (A) The probability of fixation of an allele expressing a deterministic maternal effect (blue line) or randomizing maternal effect (grey line), when invading the ancestral population exclusively composed of an allele not expressing maternal effects. Results are from 10,000 randomly drawn fitness parameters with *γ* = 2.0, thus producing approximately 2-fold fitness effects on average (see Materials and Methods). Other distributions of fitness effects yield qualitatively similar results. Fixation probabilities were calculated assuming an effective population size of 10^3^, one order of magnitude lower than experimental census sizes [53,69], and an initial frequency of 0.01, using M. Kimura’s approximation of the Wright-Fisher sampling process (see Materials and Methods) [68,70,71]. (B, C) Illustrative numerical simulations mimicking the evolution of maternal effects during experimental evolution (see Materials and Methods). (B) The probability of environmental transition was set to 0.95, as the predictable populations experienced during experimental evolution. Relative fitness values are of W_A-to-N_ = 0.335 and W_A-to-A_ = 2.63. These values are, thus, similar to those measured after experimental evolution (Fig 2C). The deterministic maternal effect allele (DME; blue line) reaches fixation at generation 24, during which the population geometric mean fitness (black line) increased to the expected value of the square root of 2.63. Fifty simulations with the same parameter values show that by generation 60 there is always fixation of the DME allele and, thus, the same geometric mean fitness among populations (black circle). The randomizing maternal effect allele (RME; grey line) does not invade the ancestral population (dashed line). (C) As in panel (B), W_A-to-N_ = 0.335 and W_A-to-A_ = 2.63, but the probability of environmental transition was 0.55, as the unpredictable populations experienced during experimental evolution. After 60 generations, the frequency of the RME allele reaches 0.91. Fifty simulations with the same parameter values show that the RME generally approaches fixation by generation 60 (grey circle; mean and one standard deviation) and, thus, that some variation is maintained in geometric mean fitness (black circle; mean and one standard deviation). Simulation code deposited in the Dryad repository: http://dx.doi.org/10.5061/dryad.56bb4 [59].

We performed a large set of simulations using environment-specific fitness parameters drawn from distributions that cover the experimentally observed fitness effects (Fig 7A). We also illustrate the evolutionary time course for one set of parameters in the predictably fluctuating regime (Fig 7B) and the unpredictably fluctuating regime (Fig 7C). We find that the randomizing maternal effect allele has a constant benefit relative to the ancestor for switching rates of less than 0.5 and is outcompeted by the deterministic maternal effect allele when the probability of switching is large. Even though the same environment-specific phenotypic effects were modelled for both maternal effects, the overall probability of fixation for the randomizing maternal effect allele is only about one-tenth that of the deterministic maternal effect allele. Under strictly alternating environments, the reduced invasion fitness and probability of fixation of randomizing maternal effects as compared with an equivalent deterministic strategy is simply due to the fact that randomizing maternal effects produce a fraction of offspring with inappropriate phenotypes for the environment they will encounter.

## Discussion

The evolution experiments and supporting theoretical modelling demonstrate that in *C*. *elegans*, maternal glycogen provisioning underlies adaptation to a temporally fluctuating anoxic environment varying across mother–offspring generations. As expected, deterministic maternal effects can be adaptive when the environmental variation over mother–offspring generations is negatively correlated, as in our predictable regime (Figs 3 and S2). Adaptation of the predictable populations (Fig 4) resulted from the evolution of a deterministic maternal effect because embryo hatchability increased when the maternal environment was normoxic (Fig 5). As a correlated response, fecundity decreased when hermaphrodites were reared under anoxia. But more directly, glycogen content in oocytes, which is known to determine later embryo survival under anoxia if mothers are reared in high-salt conditions [50,51], only increased when the mothers were reared under normoxia (Fig 6).

There was no indication that hatchability in the ancestor population was dependent on the maternal environment, only that it was more reduced under anoxia than under normoxia (Fig 2). During experimental evolution under the predictable regime, genotypes that were better able to survive anoxia were also those that were able to express the deterministic maternal effect of glycogen provisioning. It is thus interesting to compare our results with those of another evolution experiment also testing for the adaptive significance of maternal effects under predictably fluctuating environments. In the male-female nematode *Caenorhabditis remanei*, survival to a heat stress during the larval stages is reduced or improved depending on whether or not, respectively, the parental generation faces the heat stress when larvae [46]. Unlike our predictable populations, however, selection for increased larval heat shock survival every other generation for about 20 generations resulted in the loss of the ancestral paternal/maternal effect. Why the ancestral paternal/maternal effect was lost is unclear, but it may be related to the fact that the obligate outcrossing *C*. *remanei* populations had substantial standing genetic variation and, as a consequence, ancestral parent–offspring conflicts [63] or sexual conflicts [72], which needed to be resolved in order for adaptation to occur. Evidence for this scenario was that the within-generation larval heat shock plasticity in survival was also lost during experimental evolution [46,73].

In our evolution experiments, the possibility for parent–offspring and sexual conflicts were minimized, since there were hardly any males and hermaphrodites reproduced by self-fertilization (S1 Fig; [55,62]). Although the predictable populations evolved via strategic provisioning of glycogen that could be used as an energetic store to survive the anoxic stress, this finding does not rule out the possibility that the maternally transferred glycogen could also serve as a cue for initiating specific offspring gene expression. In such a case, the observed responses in the predictable populations could in part be due to the evolution of within-generation plasticity. In *C*. *elegans*, embryogenesis starts while embryos are in their mother’s uterus, and they generally arrest development and can enter a state of suspended animation under oxygen deprivation independently of maternal environment [74,75]. However, embryonic DNA transcription, RNA translation, and other metabolism can start early, maybe within one hour from fertilization [76,77], and maternal RNA, protein, and other metabolite contributions are known to be required at least until gastrulation [50,51,77,78]. Under our experimental design, embryogenesis must have ensued despite the anoxia challenge, since only hatched first-stage larvae could have initiated a new generation (Fig 1). Upon maternal glycogen provisioning, initiation of embryo glycogen metabolism to withstand anoxia is, thus, possible. Evidence for this comes from the fact that hermaphrodites from the evolved predictable populations reared under anoxia produced embryos with reduced glycogen content that did not pay a survival cost relative to the ancestor under either oxygen level treatment (Figs 4 and 6). In addition, it appears that the level of glycogen was lower in embryos than in oocytes when maternal environment is statistically corrected for (a post-hoc comparison between oocytes and embryos, not taking within-individual variation into account, shows that regardless of oxygen levels: Tukey t_461_ = 3.7, *p* = 0.002). These results suggest that embryos in utero were already metabolizing glycogen despite not having been faced with anoxia.

In the evolved predictable populations, embryos that survived anoxia were also those that were less fecund as adults (Fig 5). The fecundity response could have evolved as a correlation to the evolution of maternal glycogen provisioning, as low fecundity evidently decreases fitness. In *C*. *elegans*, compromised progeny production due to impaired oogenesis and ovulation is known to boost adult recovery and survival from oxygen deprivation [79]. It is, therefore, tempting to speculate that the fecundity–hatchability tradeoff that we measured in the predictable populations was due to increased glycogen consumption, necessary for embryo survival, limiting its allocation for oogenesis and ovulation later in adulthood. It is, of course, possible that parallel maternal and offspring-specific developmental and physiological mechanisms determined adaptation, besides glycogen provisioning and possibly consumption, which could have increased embryo survival under anoxia [50,74,79,80]. We can conclude, however, that oocyte-size-related traits were not involved, as might have been expected from classical life-history theory predicting a fitness tradeoff between offspring size and number under resource-limited situations (S7 Fig; but see [81,82]).

**Fig 8:**
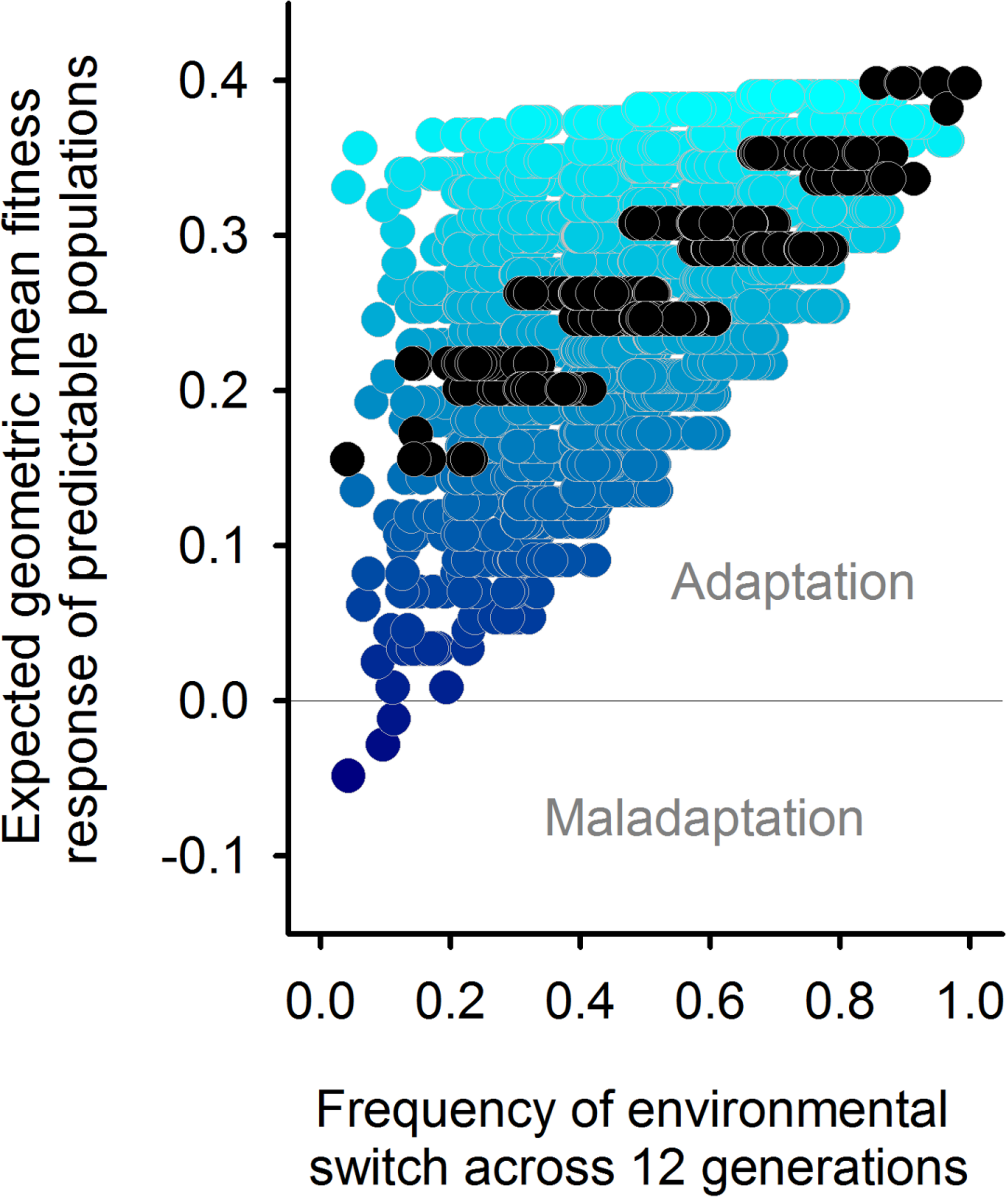
Deterministic maternal effects may underlie adaptation to a wide range of temporally fluctuating environments. Expected adaptation of the predictable populations at generation 60 relative to the ancestor state (zero line) if they were to face all possible fluctuating sequences of normoxia and anoxia for 12 generations (see Materials and Methods). Data is jittered along the *x*-axis for clarity. Each circle shows adaptation to one environmental sequence, with the dark-blue-to-cyan gradient depicting increasing frequency of normoxia generations and black circles representing sequences in which normoxia and anoxia generations are equally represented. With the exception of environmental sequences with predominant anoxia, predictable populations would adapt to a wide range of fluctuating environments due to the previous evolution of deterministic maternal effects and correlated fitness benefits.

Although hermaphrodites from the predictable populations showed improved hatchability at the expense of fecundity when faced with anoxia, under normoxia we observed an evolutionary increase in fitness, particularly when the maternal generation was also exposed to the ancestral normoxic treatment (Fig 4). This fitness response can only be explained by a correlated response to the evolution of glycogen provisioning, because the predictable populations did not experience normoxia–normoxia sequences during their history. It suggests that once maternal effects have evolved, specifically deterministic maternal effects, they might be adaptive to a range of fluctuating environments. To illustrate that deterministic maternal effects may be adaptive to a range of temporally fluctuating environments, we used the predictable populations’ two-generation fitness values (Fig 4B) to simulate the geometric mean fitness for arbitrary sequences of fluctuating oxygen levels over 12 generations (Fig 8; see Materials and Methods). This analysis shows that the evolved genotypes would perform better than the ancestral genotypes even if faced in the near future with environmental fluctuations that were not used in experimental evolution. The evolution of deterministic maternal effects and correlated fitness benefits expressed in all four maternal–offspring two-generation transitions therefore appears to have unlocked an adaptive potential that was not accessible to the ancestral population (but see [22,23,83]).

Maternal effects could have evolved as well in the constant populations [5,15], yet we found no such evidence: oocyte size, glycogen content, and hatchability remained at similar levels as in the ancestor population, and fecundity was improved irrespective of hatching environment (Fig 5). We can interpret these results in light of the life-history tradeoffs that were apparent in the predictable populations, in which evolution of enhanced hatchability and perhaps glycogen consumption resulted in the evolution of reduced fecundity (above and [79]). In the constant populations, the embryos surviving anoxia were possibly those that had more efficient glycogen metabolism and which, as a consequence, as adults had reduced fecundity. The progeny of these low-fecund hermaphrodites again faced anoxia in subsequent generations and further decreased their chances of survival until the larval stages. For populations to remain above replacement rates (Fig 3), in this scenario, selection for increased progeny production would occur and result in the observed increase in fecundity under anoxia hatching conditions (Fig 5). As a correlated response, an increase in fecundity under normoxia was also observed, showing that the selected genotypes were insensitive to their hatching conditions. Why these genotypes did not reach high frequencies under the previous laboratory adaptation to normoxia and high salt [54,55] before selection in the novel anoxia environment is likely explained by the polygenic nature of fitness components and the shifting pleiotropy patterns during evolution (S4 Fig; cf., [73,84,85]).

Our study also finds poor support that randomizing maternal effects (or developmental instability) make a strong contribution to adaptation to temporally fluctuating environments, thus failing to confirm the theoretical prediction that they would evolve when there is uncorrelated environmental variation across two generations [25,26,28,86], as in our unpredictable regime (Figs 3A and S2). Randomizing maternal effects were expected to evolve because of selection on genotypes with the ability to produce diverse broods with varying survival in anoxia (diversifying bet-hedging) or with the ability to cope with fluctuating anoxia to a sufficient degree such that environment-specific variance in fitness was reduced (conservative bet-hedging) [31,38]. We thus expected to find evolution of hatchability by the evolution of variance in oocyte and embryo glycogen content, but such was not the case. Indeed, variation in oocyte/embryo glycogen content may have been reduced at the expense of embryo hatchability (Figs 5 and S8). Parallel developmental and physiological mechanisms that we did not characterize could also have evolved, such as maternal control over the proportion of diapausing embryos [74]. But the strongest evidence against the evolution of randomizing maternal effects (or developmental instability) of any kind is the fact that the geometric mean fitness of the unpredictable populations over all the two-generation environmental transitions that they experienced may have gone down (Fig 4B) when it was expected to increase [32–36].

Finding adaptive randomizing maternal effects has been challenging [31,38,43], partly because all maternal effects have deterministic and stochastic components, and partly because randomizing maternal effects are easily confounded with within-generation phenotypic plasticity and developmental instability. However, one evolution experiment has shown that randomizing maternal effects can underlie adaptation to temporally fluctuating environments [49]. In the fungus *Neurospora crassa*, between ten and 14 rounds of fluctuating selection for ascospore germination resulted in the evolution of ascospore dormancy fraction, with populations facing uncorrelated environmental variation between selection rounds showing an increase in dormancy fraction when compared to populations facing correlated environmental variation. Nevertheless, only 12 out of the 88 replicate populations showed the expected evolutionary trends, suggesting that the likelihood of evolving randomizing maternal effects in natural conditions is low.

We have expanded elsewhere the brief theoretical analysis presented here to include direct competition between different kinds of maternal effects and parent–offspring tradeoffs under all possible patterns of environmental correlation across two generations [11]. There, we find that for randomizing maternal effects to evolve, not only must there be no environmental correlation, but also, the frequency with which different environments are present must fall within a narrow range. Importantly, fitness differences between the optimal trait values in the alternative environments must also be quite large. It is, perhaps, not surprising, therefore, that only in a few examples with extreme fitness differences, such as the evolution of seed dormancy in annual plants or the evolution of diapause in univoltine insects [24,33,86,87], have randomizing maternal effects been implicated as underlying adaptation to fluctuating environments. By contrast, deterministic maternal effects can evolve so long as the predictability of the next generation falls above a threshold level that is related to the mutual information entropy [8,11]. The numerical analysis presented here nonetheless suggests that genotypes expressing randomizing maternal effects could spread in populations experiencing the unpredictable fluctuating regime based on the observed fitness differences in anoxia and normoxia environments (Fig 7C).

Why we did not observe evolution of randomizing maternal effects in our unpredictable regime could be explained by a lack of heritability for the relevant traits in the ancestor population and subsequent poor statistical power to detect a fitness response with only eight replicate populations (Fig 7A). A nonexclusive explanation is that the evolution of transgenerational effects over more than two generations determined adaptation. Although the decrease in the geometric mean fitness of the unpredictable populations is not significant, there is a clear trend for maladaptation (Fig 4), a trend that surely resulted from decreased fecundity and hatchability under anoxia (Fig 5). Since unpredictable populations did not go extinct during experimental evolution and, indeed, showed similar expected growth rates as the predictable populations (Fig 3B), we can deduce that other unmeasured components of the long-term fitness must have increased enough to compensate the selection against fecundity and hatchability over one or two generations of anoxia. Directly showing these long-term fitness components is beyond the scope of the present study, since increasing the number of generations considered results in a geometric increase in the number of assays required. Theory has shown, however, that multigeneration carryover effects can be selected in fluctuating environments [16–18,88–90], and in *C*. *elegans*, transgenerational effects over more than two generations have been described [91–93]. In our experiments, there was room for the evolution of these transgenerational effects, since the unpredictable populations faced environmental variation that was moderately correlated at multigeneration intervals (S2 Fig).

In summary, our study demonstrates that maternal metabolic resource allocation toward offspring protection can evolve and determine adaptation to temporally fluctuating environments. Our study finds much less support that bet-hedging strategies can underlie adaptation to temporally fluctuating environments.

## Materials and Methods

### Experimental Evolution Design

All populations were derived from a population gradually adapted for 50 generations to high salt (305 mM NaCl, GA250) [55], which, in turn, was derived from a population adapted during 140 generations to our standard laboratory life cycle and demography (A6140) [53–55]. Frozen GA250 samples with >10^4^ individuals were thawed from frozen -80°C stocks and cultured for one generation for number expansion, before replicate population derivation. Predictable, unpredictable, and constant populations were designated Pi, Ui, and Ci, respectively, with *i* standing for replicate number (see next section below). During experimental evolution, a random number was assigned to each population to avoid potential manipulation bias. Periodic cryogenic storage at -80°C was done during experimental evolution [94]. Following our standard laboratory life cycle and demography (Fig 1) [54,55], each population was kept in ten 9-cm Petri plates with 28 mL of solid NGM-lite agar media (Europe Bioproducts) covered by an overnight grown lawn of HT115 *Escherichia coli* that served as ad libitum food. NaCl concentration in the NGM-lite media was 305 mM (1.78% w/v) [55]. At 24 h ± 2 h of the life cycle, each population was seeded with 1,000 synchronized first larval staged (L1) individuals in each of the ten Petri plates. After growth to maturity for 66 h ± 2 h at constant 20°C and 80% RH in controlled incubators (Fitoclima D1200, ARALAB), all ten plates were mixed, and worms were harvested with 5 mL M9 isotonic solution and then exposed to 1M KOH: 5% NaOCl “bleach” solution for 5 min ± 15 s, to which only embryos survive [54,94]. After repeated washes with M9, 200 μL containing embryos (and larval and adult debris) were transferred to 25 mM NaCl NGM-lite plates, without *E*. *coli*. A large faction of the adults is not disrupted after the bleach solution treatment and, thus, in utero embryos can continue their normal development despite the bleach solution. These embryos are unlikely to contribute to the next generation, however, as they would need to break the adult body wall in order to be passaged (see below). The Petri plates were then placed inside 7L polycarbonate boxes with rubber-clamp-sealed lids (AnaeroPack, Mitsubishi Inc.). Within these boxes, an anoxic embryo hatching condition was imposed by placing two GasPak EZ sachets (Becton, Dickinson and Company). These sachets contain inorganic carbonate, activated carbon, ascorbic acid, and water, which after 2 h ± 1 h of activation will produce an anaerobic/anoxic atmosphere inside the boxes (<1% O_2_, ≥13% CO_2_; according to the manufacturer). At every generation, in all boxes, anoxia conditions were confirmed by placing two BBL Dry Anaerobic Indicator strips (Becton, Dickinson and Company). To prevent drying, paper towels with 20 mL ddH_2_0 were placed within each box. For normoxia hatching conditions, the sachets were not used, and an additional 60 mL ddH20 was placed inside each box. After 16 h ± 1 h, boxes are opened and synchronized starvation-arrested live L1s were washed off the plates with 3–5 mL M9 to a 15 mL Falcon tube, adult debris removed after centrifugation at 200 rpm, and density estimated under a Nikon SMZ1500 dissection scope in five 5 uL M9 drops at 40x magnification. We never observed L2 or L3 larval staged individuals at this stage—those that could potentially have broken their mothers’ body wall during normoxia/anoxia treatment—suggesting that embryos in the uterus that continued their development after bleach solution exposure did not significantly contribute to the next generation. Instead, those that contributed to the next generation were already laid embryos before exposure to the bleach solution. Synchronized L1 density during experimental evolution provided the expected growth rate data shown in Fig 3B. While estimating, Falcon tubes were kept in a shaker at 120 rpm (Lab. Companion SK-600) inside the incubators for aeration. 6 h ± 1 h after, the appropriate M9 volume for 1,000 live L1s was placed in fresh NGM-lite plates to complete one life cycle.

### Normoxia–Anoxia Temporal Regimes during Experimental Evolution

QBasic64 v0.954 was used to design several environmental sequences that were assigned random numbers. The predictable environmental sequence was designed such that the probability of repeating the same oxygen level hatching condition at lag two was 0.05 over 59 mother–offspring transitions, and the frequency of anoxia and normoxia events was 0.5 over the total of 60 generations (Figs 3A and S2). Four replicate populations were cultured under the predictable regime (P1-4). Unpredictable environmental sequences were designed such that the probability of repeating the same oxygen level hatching conditions at lag two was 0.46 over 59 mother–offspring transitions, with the frequency of anoxia and normoxia generations being 0.5 over a total of 60 generations. These unpredictable environmental sequences were further designed such that the probability of repeating the same oxygen level hatching conditions at lag three were 0.52 (sequences #11, #19), 0.34 (sequence #25), and 0.62 (sequence #31) over 58 grandmother–offspring transitions. Two replicate populations were cultured under each of these four unpredictable conditions (U1-2, U3-4, U5-6, and U7-8, respectively, for sequences #11, #19, #25, and #31). The constant environmental sequence was characterized by 30 consecutive generations of anoxia followed by 30 generations of normoxia (Fig 3A). In S2 Fig, we show the autocorrelation and spectral decomposition of anoxia–normoxia fluctuations at several generation intervals of all the sequences employed.

### Growth Rate Assays

P1-4 and U1-8 from generation 60 and C1-4 from generation 30 were measured for expected growth rates alongside the ancestral GA250 population, in two-generation assays encompassing all four mother–offspring oxygen hatching environment combinations (Fig 1). C1-4 from generation 60 were further assayed in anoxia–anoxia or normoxia–normoxia two-generation assays. For all assays, frozen -80°C stocks (*n* > 10^3^) were thawed and reared in a common environment for two generations before assaying. On the third generation, adults were washed off the plates, treated with the “bleach” solution, and their embryos were exposed to normoxia or anoxia to constitute the maternal hatching assay environment. 24 h later, 1,000 surviving L1s were seeded in each of five Petri dish plates, allowed to grow to adulthood, treated with the “bleach” solution, and their embryos were exposed in a factorial fashion to normoxia or anoxia to constitute the offspring hatching assay environment. After 16 h of exposure to the corresponding hatching environment, worms were washed off the plates with 3–5 mL of M9 to a 15 mL Falcon tube before the total number of surviving L1s was estimated by considering the total volume of the M9 solution and counting the number of live L1s in ten to 15 5 μL drops. The total number of live L1s was then divided by 5,000 to calculate the maternal L1 to offspring L1 growth rate. Since the assay encompasses a full life cycle mimicking the conditions of experimental evolution, the observed growth rates are estimates of absolute fitness. The assays were done in 18 blocks, defined by different thawing dates of the samples. In each block, the ancestral GA250 population was included. There were 3 measurements per population and hatching treatment, with replicates being independently maintained since the maternal setup generation.

### Statistical Analysis of Relative Fitness Data

Several functions within the packages *stats*, *lme4*, *lsmeans*, and *pkbrtest* in R were used for computation [95]. The natural logarithm of the ancestral growth rate data from all blocks was analyzed with LMM and REML estimation methods [56], taking maternal and offspring hatching treatment as two separate fixed factors, each with two levels (normoxia or anoxia), and block as a random factor. We took the natural logarithm of the growth rate data for modelling, since the scale of normoxia and anoxia data are very different, leading to the residuals of the model being non-normally distributed (as tested with Shapiro-Wilk test). Planned contrasts were performed among maternal hatching conditions and among offspring hatching conditions within maternal condition, using post-hoc Tukey *t* tests [57], while estimating the “effective” degrees of freedom for these tests with the KR approximation [58]. We show in Fig 2A the least-square mean and error estimates from the LMM, with positive values implying that the population would grow in numbers if allowed to.

To analyze the growth rate responses during experimental evolution (Fig 3C), we modelled the natural logarithm of the ratio of the growth rate estimates of the evolved populations (generation 60 P1-4 and U1-8, or generation 30 C1-4) over the ancestral mean value per block. By taking the ratio of the evolved observations over the mean ancestral value per block, we are therefore able to quantify heterogeneity among replicate populations. Further, this ratio is an estimate of the relative fitness difference between derived and ancestral populations, with positive values implying adaptation. If adaptation is due to a single codominant allele (or, analogously, a haploid complement), the natural logarithm of the ratio of evolved over ancestral observations estimates selection strength [70,96]. Using LMM, we took the maternal–offspring hatching conditions as a single fixed factor with four levels and replicate population as the random factor to account for the effects of genetic drift and other historical accidents unique to each evolved population. Each experimental evolution regime was modelled separately, since the replicate population effects could be under or overestimated if all regimes were included in the same model. Specifically, unpredictable populations were 8-fold replicated in four different environmental sequences (and not 4-fold replicated in one environmental sequence as the other experimental regimes), and the extent of genetic drift among the constant generation 30 populations was at least halved relative to the other populations from generation 60 (Figs 3B and S4). Significant evolutionary responses were inferred when the least-square mean estimates from the model were different from zero, the ancestral value. For this, we used post-hoc two-tailed Student *t* tests with LMM-corrected KR degrees of freedom taking into account the overall LMM error. All models’ residuals were checked for normality with Shapiro-Wilk tests. In the case of the predictable regime model, residuals were non-normal due to three outlier observations. Models with or without outliers gave similar results, however. In the case of the constant regime model, residuals were non-normal with ln-transformed data but normal in the untransformed scale. Both models gave similar results.

Constant populations were similarly modelled for evolutionary responses at generations 30 and 60, in anoxia–anoxia or normoxia–normoxia maternal–offspring conditions (S3 Fig). LMM was used to model ln relative fitness by taking generation and maternal–offspring treatment as fixed factors and replicate population as a random factor. Besides testing for responses relative to the ancestor, we also tested for differences between generations within each maternal–offspring treatment, again with two-tailed Student *t* tests using the KR corrected degrees of freedom.

For predictable and unpredictable regimes at generation 60, we obtained the least-square mean relative fitness per replicate population and per maternal–offspring treatments (LMM with block as random factor) to calculate the geometric mean fitness across the maternal–offspring treatments that each faced during experimental evolution. We then tested in each regime whether or not there was a significant response in ln geometric mean fitness relative to the ancestor with Student *t* tests.

### Fecundity and Hatchability Assays

These assays were similar in design to the fitness assays, with two-generation exposure to all four mother–offspring oxygen hatching environment combinations. P1-4 or U1-4 from generation 60 or C1-4 from generation 30 and the ancestor GA250 (*n* > 10^3^) were thawed and grown in parallel for two generations before assaying. For each replicate measurement, 1,000 live L1s after exposure to normoxia or anoxia maternal hatching environments were grown in six to ten plates each. Adult worms were washed off and treated with the “bleach” solution. In contrast to the fitness assays, after the “bleach” treatment, the dead adults were removed from the M9 Falcon tube after centrifugation at 200 rpm. The number of embryos was then immediately scored in ten 5 μL M9 drops. This total number was then divided by the total number of adults to calculate a per capita fecundity. Note that survivorship differences between the L1 larval stage and adulthood are encompassed by this definition of fecundity (Fig 1). The embryo-only M9 solution was equally divided within 2 h after the “bleach” treatment and centrifuged at 1,800 rpm. The pellet containing the embryos was then exposed to anoxia or normoxia hatching conditions. After 16 h, the density of live L1s was estimated. Per capita hatchability was calculated as the ratio of live L1s over embryo number. The fecundity and hatchability assays were done in eight thawing time blocks for three independent measurements per population and hatching condition since the maternal assay setup generation.

### Statistical Analysis of Fecundity and Hatchability Data

Ancestral data from all blocks was modelled with LMM by taking maternal hatching treatment as a fixed factor with two levels in the case of fecundity data (Fig 2B), and maternal and offspring hatching treatments as two fixed factors, each with two levels in the case of the hatchability data (Fig 2C). Block was modelled as a random factor. To keep analytical consistency between all traits measured, we modelled the natural logarithm of the ratio of the fecundity or hatchability estimates of the evolved populations (generation 60 P1-4 and U1-4, or generation 30 C1-4) over the ancestral mean value per block. Note, however, that the ln of the evolved-over-ancestral ratio estimates the effective selection coefficient of a genotype that has increased in frequency exponentially, from initially low frequencies [96]. Each regime was separately modelled with LMM, taking maternal hatching treatment as a fixed factor with two levels in the case of fecundity data (Fig 5A), and maternal–offspring hatching treatments as a fixed factor with four levels in the case of the hatchability data (Fig 5B). Replicate populations were modelled as the random factor. As before, to infer significant evolutionary responses, we used two-tailed Student *t* tests with the LMM-corrected KR degrees of freedom to test for differences to zero.

### Oocyte and Embryo Glycogen Content and Oocyte Size Assays

To measure glycogen content, we followed standard procedures involving iodine vapor staining of individual hermaphrodites [50,51,97]. Hermaphrodites from generation 60 P1-4 or U1-4, or from generation 30 C1-4, and the ancestral GA250 populations were assayed for glycogen content in the oocytes and embryos in utero, after samples were thawed and grown in parallel for two generations before assaying. On the third generation, and after exposure to a normoxia or an anoxia hatching environment, day four adult hermaphrodites were subjected to fixation. This was done by suspending them in a solution of 700 μL of absolute ethanol (VWR Scientific) with 200 μL of glacial acetic acid (Carlo Erba) and 100 μl of concentrated formalin (Sigma-Aldrich) for 90 min. Serial dehydration was then done by sequentially resuspending worms in 70%, 90%, and absolute ethanol. Samples were stored at -20°C. Following standard protocols [50,52,97], frozen hermaphrodites were sequentially rehydrated in absolute, 90%, and 70% ethanol, before being resuspended in the M9 solution. Hermaphrodites were then transferred to a glass slide topped with a thin agar pad (5% noble agar; Becton, Dickinson and Company) with a pipette. Hermaphrodites from the ancestral and one of the evolved populations were assayed simultaneously by placing them on the same agar pad. For staining, each glass slide was placed upside down over the mouth of a bottle with 100g of iodine (I_2_, ACS ≥ 99.8% solid; Sigma-Aldrich) for 120 s. Within 40 mins, photographs were taken at 630x magnification under differential interference contrast (DIC) settings in a Zeiss Axioskop2 microscope coupled to a monochromatic CCD camera (Hitachi Denshi ltd.). The DIC settings for the microscope and camera were kept identical across all glass slides. Two to three images were taken to cover up to 20 developing in utero embryos and/or the first three oocytes posterior from the spermatheca/uterus, in each of three to eight individual hermaphrodites. Similar images were obtained for unstained samples from GA250 and P1-4 from generation 60. To estimate within-hermaphrodite variation in glycogen content in GA250 and U1-4 from generation 60, images were taken from both the anterior and posterior gonads covering three to five oocytes. The images were not manipulated for contrast or grayscale levels. For analysis, we used ImageJ 1.46r, with the first three oocytes from the spermatheca, or all visible embryos, each being manually delineated and the mean pixel intensity over the area recorded (S5 Fig). For all the images, the pixel intensity of agar pad on either side of the worm was also obtained to account for the decay of staining with time and/or other nonspecific staining variation across images. The ratio of the mean pixel intensity of the oocytes and/or embryos over the mean pixel intensity of the agar pad per glass slide was taken as the proxy for glycogen content (see S5 Fig, for the observed values in the ancestral population). Size was taken as the perimeter in pixels of the individually delineated oocytes. Size of the embryos was not considered due to the uncertainty in delineating them. In any case, embryo size is mostly determined by oocyte size. The assays were run in eight blocks with measurements taken over 24 slides.

### Statistical Analysis of Glycogen Content and Oocyte Size Data

As for the other traits, we modelled the natural logarithm of the ratio of glycogen content of the derived populations (generation 60 P1-4 and U1-4, or generation 30 C1-4) over the ancestral mean value per glass slide. Each regime was separately modelled with LMM, taking maternal hatching treatment as a fixed factor with two levels and random population and individual hermaphrodite as random factors (Fig 6). For oocyte and embryo glycogen content data in the predictable populations, LMM residuals deviated from normality, but results are similar when removing two or three “outlier” observations, respectively, to resolve this issue. Ancestral and generation P1-4 populations were also scored for pixel intensity in slides that were not stained with iodine, to confirm that the responses observed are specific to stained samples. Similar LMM was done on unstained samples. Variance component analysis of glycogen content in the ancestral and unpredictable populations is shown and explained in S8 Fig.

### Genotyping

Immature L4 hermaphrodites were handpicked from GA250, generation 60 P1-4 and U1-4, and generation 30 and generation 60 C1-4 populations, and their gDNA collected using the prepGEM Insect kit (ZyGEM). Biallelic SNPs were chosen based on the pooled genome sequence of the A6140 population (M. Rockman and H. Teotónio, unpublished data). SNPs were genotyped with the iPlex Sequenom technology [98]. WS200 genome version was used for the oligonucleotide design and physical coordinates (www.wormbase.org). SNPs in chromosome I and II were genotyped in 48 GA250 individuals and in 16 individuals from each of the evolved populations. Chromosomes III and IV and, separately, V and VI were genotyped with similar sample sizes. For data quality control, SNPs had to be polymorphic in A6140. SNPs for which the assay failed in more than 30% of the samples were eliminated. This was followed by elimination of individuals with more than 10% undetermined allele identity and, finally, elimination of SNPs with failed assays in more than 15% of the remaining individuals across populations. A total of 405 SNPs were used for analysis at approximate densities of 4.3 SNPs/Mbp (chr. I), 5.6 SNPs/Mbp (chr. II), 5.3 SNPs/Mbp (chr. III), 2.8 SNPs/Mbp (chr. IV), 4 SNPs/Mbp (chr. V), and 6 SNPs/Mbp (chr. VI).

### Genetic Variation Analysis

For the ancestor population GA250, within-population fixation indices (Fis), a measure of inbreeding by selfing, were calculated as 1 minus the ratio of observed (Ho) to expected heterozygosity levels under random mating (S1 Fig) [60]. Haplotype reconstruction was done using *fastPHASE 1*.*2* [99]. For each population sample, 20 random starts of the EM algorithm were employed with 200 haplotypes taken from posterior distributions. The number of clusters for cross-validation was set to 10, and SNPs with posterior probabilities below 0.9 were considered missing data. Effective number of haplotypes was calculated as *1/Σp*_*i*_^*2*^, with *p*_*i*_ being the proportion of haplotype *i* across all reconstructed haplotypes (S4 Fig) [60].

### Mathematical Modelling

All computation was done using Wolfram Mathematica 9. We took two approaches, one based on a general model of maternal effects that we used to predict when they are likely to evolve, and another based on the empirically observed relative fitness responses that we used to predict how the evolved populations would respond to novel environmental sequences. In both cases, evolutionary theory shows that when there is generation-to-generation variance in reproductive output, the geometric mean of fitness is the appropriate measure to quantify the expected genotype frequency change [32,33]. The presence of maternal effects does not change this principle, but does require considering the frequency of each two-generation environmental transition.

In the general model, we calculated the geometric mean fitness of genotype *k* as *G*_*k*_ = exp(∑ *p*(*i*,*j*)log(*w*_*k*_ (*i*,*j*))), where *G*_*k*_ represents the geometric mean fitness of genotype *k*, *p*(*i*,*j*) is the frequency of transitions from hatching environment *i* to hatching environment *j*, and *w*_*k*_ (*i*,*j*) is the reproductive output of a genotype *k* individual developing in environment *j* whose mother experienced hatching environment *i*. We model maternal effects by assuming that there are two possible offspring phenotypic states: normoxia adapted and anoxia adapted. Without loss of generality, we assigned the normoxia-adapted phenotype a relative fitness value of 1, so that *w*_*N*_ (*i*,*j*) = 1. We assigned the log fitness of the other anoxia phenotype by drawing from an exponential distribution with parameter *γ* so that fitness in anoxia was increased and fitness in normoxia was decreased. A value of *γ* = 2.0 means that the average advantage of the anoxia adapted phenotype in anoxia conditions is about 2-fold, on the order of the fitness increase we observed following experimental evolution. For simplicity, but without loss of generality, the ancestral phenotype, the normoxia-adapted phenotype, had higher geometric mean fitness than the anoxic phenotype.

We then used the geometric mean to calculate the probability of fixation of either a genotype conferring a Deterministic Maternal Effect (DME) or a Randomizing Maternal Effect (RME) (i.e., bet-hedging). The ancestral state was assumed to have no maternal effect and, therefore, produce a constant phenotype adapted to normoxia conditions. Under DME, mothers alter their offspring phenotype based on their own hatching environment. Under RME, mothers produce a fraction (*q*) of offspring with the anoxia-adapted phenotype and *(1-q)* with the normoxia phenotype. We assumed *q* was tuned to maximize the geometric mean fitness of the genotype, an assumption that favors RME. Given that experimental populations were maintained in discrete time non-overlapping generations, the probability of fixation of an invading genotype (labelled *U*_*DME*_ and *U*_*RME*_, respectively) was calculated using M. Kimura’s approximation of the Wright-Fisher process (Fig 7A) [35,100]. In line with other evolution experiments, we further assumed that effective population sizes were one order of magnitude lower than the experimental census sizes [53,69]. It is likely that our populations followed Wright-Fisher processes of sampling [70]. We defined the effective selection coefficient for use in Kimura’s equation as the geometric mean of the reproductive output of a genotype, which is expected to be a good approximation so long as the probability a genotype goes extinct due to selection is not abnormally high in early generations, e.g., [35,68,71]. Because our populations had standing genetic variation, we wished to calculate the probability that DME or RME would become common based on both strategies being present at some low initial frequency. We therefore adjusted our calculations to include the chance that both strategies would become established at appreciable frequencies, in which case the strategy with higher geometric mean fitness is expected to prevail. Thus, for parameter values that give DME a higher geometric mean, this is simply *U*_*DME*_. However, if RME had a higher geometric mean, this becomes *U*_*DME*_(1 − *U*_*RME*_).

We also performed stochastic simulations of the spread of DME and RME genotypes into an ancestral population composed mostly of genotypes that produce only the normoxia-adapted phenotype (Fig 7B and 7C). We used a population size of 1,000 and an initial frequency of both ME strategies of 0.005. RME strategies were assumed to use the optimal frequency of producing normoxia- or anoxia-adapted phenotypes. Again, we used the Wright-Fisher model to simulate changes in genotype frequency. In each generation, the relative number of offspring produced by each genotype was simply the frequency of genotype *i* multiplied by the average fitness of genotype *i* in the current environment. The number of individuals of genotype *i* was updated by sampling from a multinomial distribution in which the probability of drawing genotype *i* was the reproductive output of genotype *i* divided by the total reproductive output of the population. We also calculated the population level fitnesses as they would have been measured in our experimental assays and used these to calculate the geometric mean fitness of the population.

For our second modelling approach, we constructed lists of sequences of 12 generations of normoxia or anoxia and then used the assayed fitness values to calculate the “expected adaptation” of the evolved predictable populations relative to the ancestral population (Fig 8). The total frequency change of a genotype over multiple generations is a simple function of the product of the relative fitness of the two genotypes in each generation. We therefore defined the expected adaptation as $A_{s}=(\prod_{t=1}^{12}w_{t})$, where the relative fitness of the novel genotype is *w*_*t*_ in generation *t*, and *A*_*s*_ measures the per-generation fitness advantage of the novel genotype in an environmental regime characterized by sequence *s*.

### Archiving

Experimental design, data, and simulation scripts are deposited in the Dryad repository: http://dx.doi.org/10.5061/dryad.56bb4 [59].

## Supporting Information

S1 Fig Standing genetic variation in the ancestral population. Observed heterozygosity (*Ho*) and fixation indices (*Fis*) of 405 SNPs ranked according to their minor allele frequency (*maf*) in the high-salt-adapted population (see Materials and Methods) [55]. High *Fis* values indicate a high level of inbreeding [60], due to a high proportion of hermaphrodites reproducing by self-fertilization. The proportion of males in this ancestor population was approximately 5% [55]. During the evolution experiments reported here, no males were observed. Data deposited in the Dryad repository: http://dx.doi.org/10.5061/dryad.56bb4 [59].
(TIF)

S2 Fig Environmental sequences employed during experimental evolution. Left plot shows the autocorrelation function of the 60 generation environmental sequences employed until an interval of 16 generations. Right plot shows the spectral decomposition of environmental fluctuations (as the cumulative periodogram, see pp. 392–397 in [56]), also until an interval of 16 generations. A white spectrum, in which there is no bias in the amplitude of environmental fluctuations across time, is shown as a dashed line. In the constant regime (orange), a positive autocorrelation gradually decays to zero. The amplitude of environmental fluctuations tends to be higher at longer intervals (red spectrum). In the predictable regime (blue), the autocorrelation fluctuates between negative values at odd generations and positive values at even generations, the magnitude of which gradually decays to zero. The amplitude of environmental fluctuations tends to be higher at shorter timespans (blue spectrum). In the unpredictable regimes (greys), two sequences show little autocorrelation, while one shows moderate positive autocorrelation at lag 2, and another shows moderate negative autocorrelation at lag 2. Unpredictable environmental sequences tend to show white to light-blue spectra. The *acf* and *spectrum* functions in the *stats* package in R were used for calculation [56]. Environmental sequences are deposited in the Dryad repository: http://dx.doi.org/10.5061/dryad.56bb4 [59].
(TIF)

S3 Fig Standing genetic variation after experimental evolution. Stacked bars of SNP counts by 0.05 bins of minor allele frequency (*maf*, left) or the mean effective haplotype number found in chromosome I-II, III-IV, and V-VI with associated standard error of the mean among replicate populations (right). Results are shown for the ancestral population, generation 60 predictable and unpredictable populations, and generation 30 and generation 60 constant populations. See Materials and Methods for assay design and statistical details. Data deposited in the Dryad repository: http://dx.doi.org/10.5061/dryad.56bb4 [59].
(TIF)

S4 Fig Adaptation under the constant regime. Relative fitness of constant populations at generation 30 and generation 60 to the ancestor population (zero line), across the two combinations of maternal–offspring hatching environments they experienced during experimental evolution. Ancestor and evolved populations were concurrently assayed to account for assay block effects (see Materials and Methods). Mean and error least square estimates are shown after LMM, taking replicate population as a random factor and generation and maternal–offspring hatching treatment as fixed factors. Significant relative fitness responses tested with Student *t* tests and LMM-corrected KR degrees of freedom are shown above each bar; post-hoc Tukey *t* tests with LMM-corrected KR degrees of freedom are shown among generations: * *p* < 0.05; ** *p* < 0.01; *** *p* < 0.001. Data deposited in the Dryad repository: http://dx.doi.org/10.5061/dryad.56bb4 [59].
(TIF)

S5 Fig Glycogen content in the ancestral population. (A, B) Glycogen content is quantified in iodine-stained hermaphrodites at the time of usual reproduction during experimental evolution, following [50,52]. Illustrative photographs of stained hermaphrodites with oocytes (A) and unstained hermaphrodites with in utero embryos (B) are shown from ancestral hermaphrodites. The width of the black scale bar is 10 μm. The ratio of the mean pixel intensity of all the delineated oocytes or embryos (yellow lines) and the mean pixel intensity over the agar pad (white lines) was used for analysis (C and D, respectively). Data deposited in the Dryad repository: http://dx.doi.org/10.5061/dryad.56bb4 [59].
(TIF)

S6 Fig Evolution of unstained oocyte and embryo controls. Oocyte (A) and in utero embryo (B) unstained hermaphrodite response of predictable populations at generation 60, relative the ancestor population (zero line). Except for iodine-staining, all other assay details were the same as those presented in Figs 6 and S5. Mean and error least square estimates are shown after LMM, taking replicate population and individual hermaphrodite as random factors and maternal hatching treatment as a fixed factor. Significant evolutionary response (Student *t* test, with LMM-corrected KR degrees of freedom) is shown above one of the bars: * *p* < 0.05. Data deposited in the Dryad repository: http://dx.doi.org/10.5061/dryad.56bb4 [59].
(TIF)

S7 Fig Evolution of oocyte size. From the glycogen content assay, three oocytes were measured as the perimeter in pixels within hermaphrodites (white lines in S5A Fig). Shown are the absolute measurements of the ancestral population (A) and the oocyte size response in populations from all experimental regimes (B). Mean and error least square estimates are shown after separate LMM for each regime, taking replicate population and individual hermaphrodite as random factors and maternal hatching treatment as a fixed factor. There were no evolutionary responses, except perhaps in the unpredictable populations under maternal normoxia hatching (Student *t* test with LMM-corrected KR degrees of freedom: t_8.6_ *p* = 0.08). Data deposited in the Dryad repository: http://dx.doi.org/10.5061/dryad.56bb4 [59].
(TIF)

S8 Fig Evolution of variation in glycogen content within broods. Percent of total variation explained by variation in oocyte glycogen content (A) or in utero embryo glycogen content (B). Separate LMMs were done per maternal hatching environment and regime. In the ancestral population, individual oocytes or embryos were modelled as a random factor nested within a random glass slide. In the unpredictable regime, individual oocytes or embryos were modelled as a random factor nested within a random glass slide nested within a random replicate population. Data had to be ln-transformed so that the models were identifiable and the algorithm reached convergence. Given unequal sample sizes per slide and, by design, different numbers of populations in each regime, results should be interpreted with caution, though there is no trend for an increase in brood trait variation, as would be expected with the evolution of a randomizing maternal effect. Data deposited in the Dryad repository: http://dx.doi.org/10.5061/dryad.56bb4 [59].
(TIF)

## Abbreviations

KR: Kenward-Roger
LMM: linear mixed-effect models
SNP: single-nucleotide polymorphism
