# Supplementary figures and images for: Adaptation to Temporally Fluctuating Environments by the Evolution of Maternal Effects
Source: PLoS Biol. 2016 Feb 24;14(2):e1002388. doi: 10.1371/journal.pbio.1002388 (PMC4766184; doi:10.1371/journal.pbio.1002388)

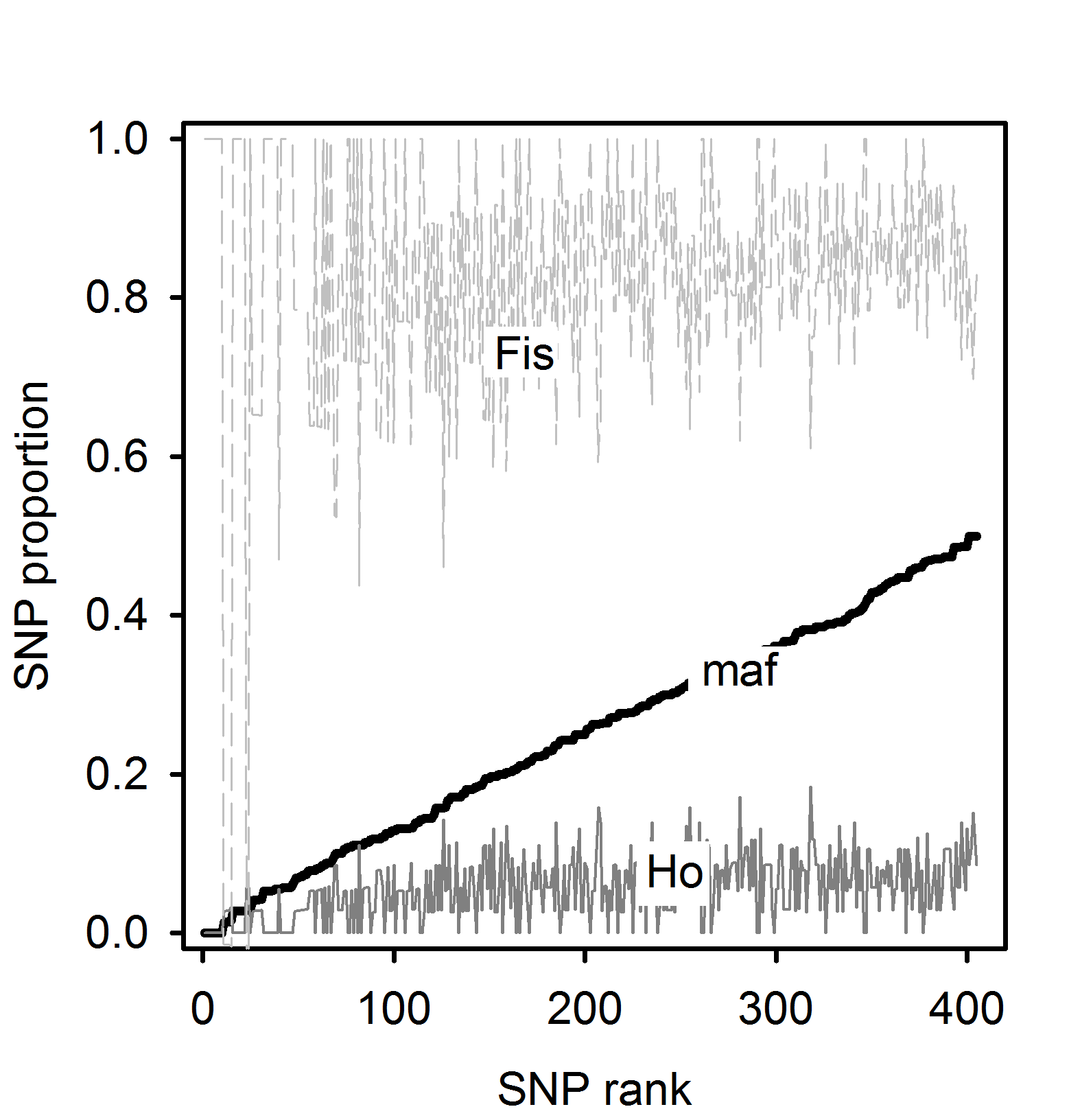

Supplement: S1 Fig — Observed heterozygosity (Ho) and fixation indices (Fis) of 405 SNPs ranked according to their minor allele frequency (maf) in the high-salt-adapted population (see Materials and Methods) [55]. High Fis values indicate a high level of inbreeding [60], due to a high proportion of hermaphrodites reproducing by self-fertilization. The proportion of males in this ancestor population was approximately 5% [55]. During the evolution experiments reported here, no males were observed. Data deposited in the Dryad repository: http://dx.doi.org/10.5061/dryad.56bb4 [59]. (TIF) [file pbio.1002388.s001.TIF]

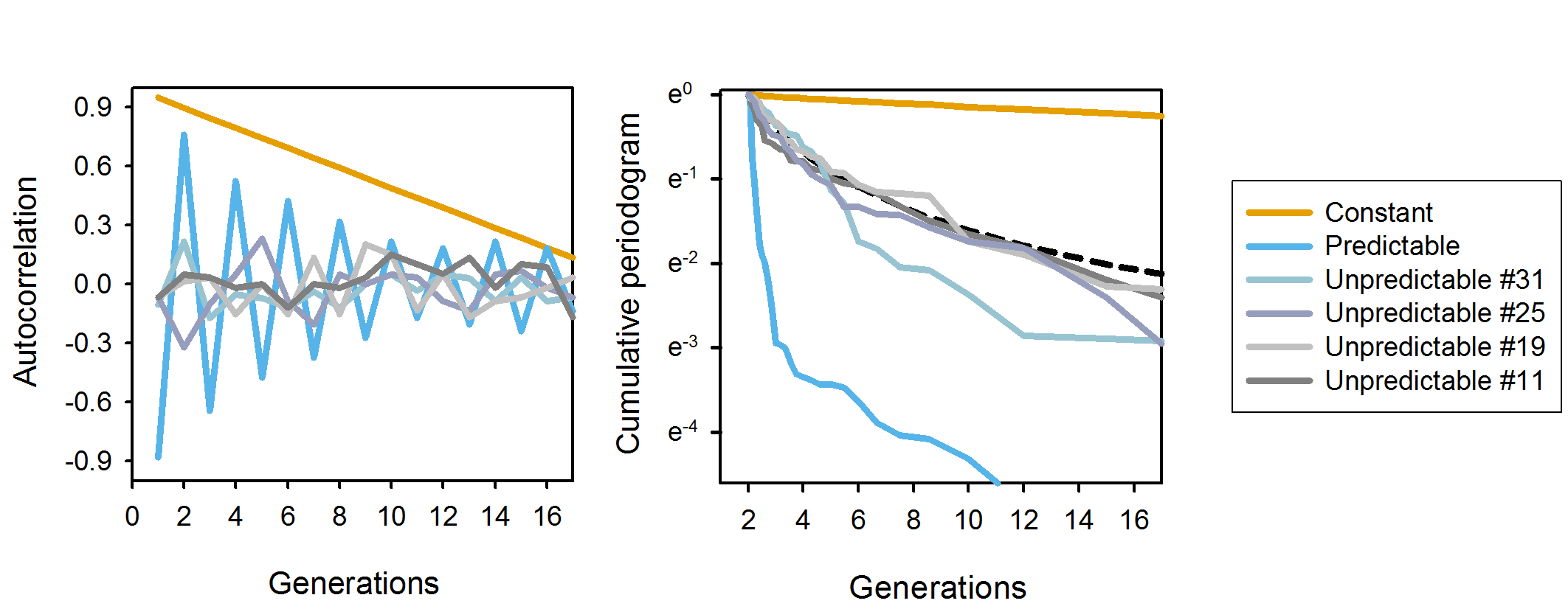

Supplement: S2 Fig — Left plot shows the autocorrelation function of the 60 generation environmental sequences employed until an interval of 16 generations. Right plot shows the spectral decomposition of environmental fluctuations (as the cumulative periodogram, see pp. 392–397 in [56]), also until an interval of 16 generations. A white spectrum, in which there is no bias in the amplitude of environmental fluctuations across time, is shown as a dashed line. In the constant regime (orange), a positive autocorrelation gradually decays to zero. The amplitude of environmental fluctuations tends to be higher at longer intervals (red spectrum). In the predictable regime (blue), the autocorrelation fluctuates between negative values at odd generations and positive values at even generations, the magnitude of which gradually decays to zero. The amplitude of environmental fluctuations tends to be higher at shorter timespans (blue spectrum). In the unpredictable regimes (greys), two sequences show little autocorrelation, while one shows moderate positive autocorrelation at lag 2, and another shows moderate negative autocorrelation at lag 2. Unpredictable environmental sequences tend to show white to light-blue spectra. The acf and spectrum functions in the stats package in R were used for calculation [56]. Environmental sequences are deposited in the Dryad repository: http://dx.doi.org/10.5061/dryad.56bb4 [59]. (TIF) [file pbio.1002388.s002.TIF]

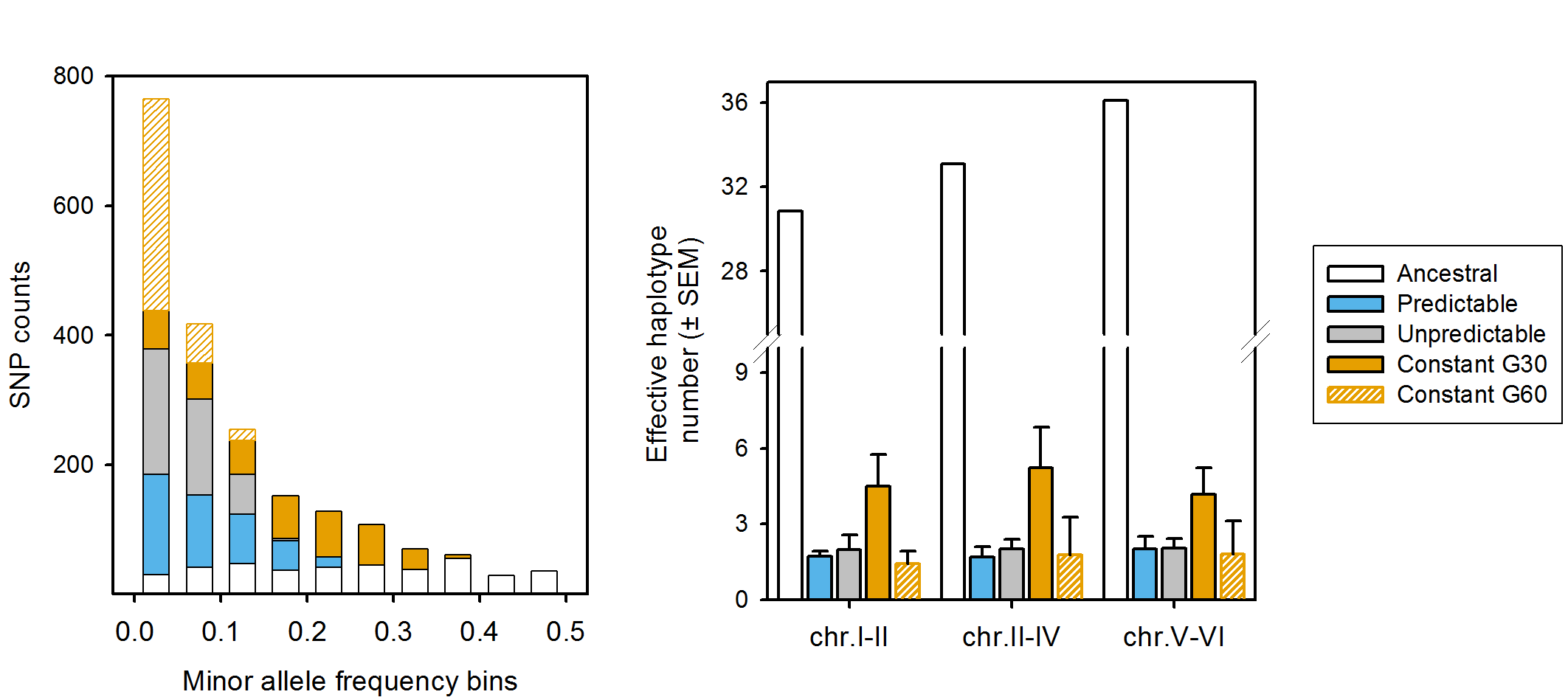

Supplement: S3 Fig — Stacked bars of SNP counts by 0.05 bins of minor allele frequency (maf, left) or the mean effective haplotype number found in chromosome I-II, III-IV, and V-VI with associated standard error of the mean among replicate populations (right). Results are shown for the ancestral population, generation 60 predictable and unpredictable populations, and generation 30 and generation 60 constant populations. See Materials and Methods for assay design and statistical details. Data deposited in the Dryad repository: http://dx.doi.org/10.5061/dryad.56bb4 [59]. (TIF) [file pbio.1002388.s003.TIF]

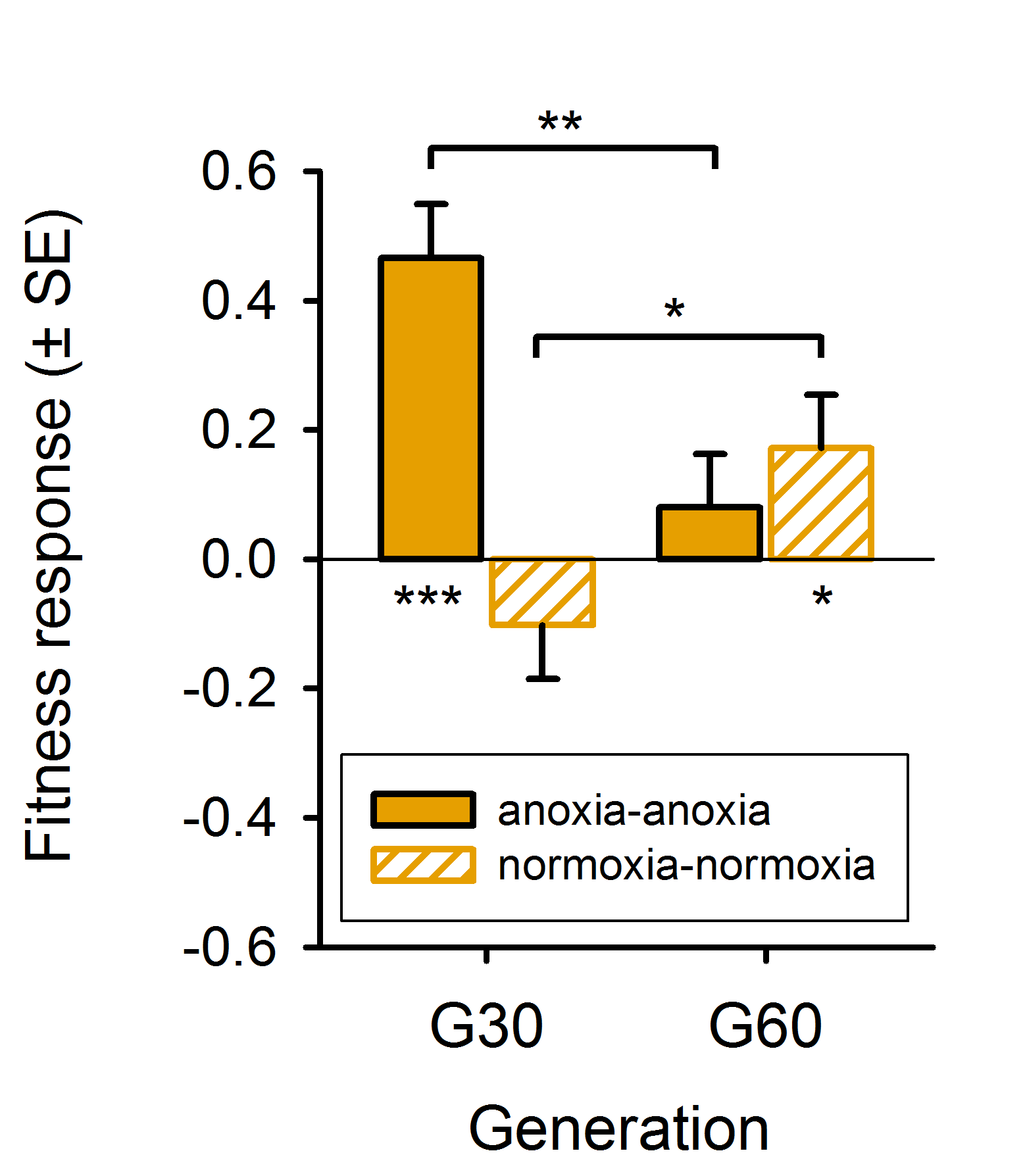

Supplement: S4 Fig — Relative fitness of constant populations at generation 30 and generation 60 to the ancestor population (zero line), across the two combinations of maternal–offspring hatching environments they experienced during experimental evolution. Ancestor and evolved populations were concurrently assayed to account for assay block effects (see Materials and Methods). Mean and error least square estimates are shown after LMM, taking replicate population as a random factor and generation and maternal–offspring hatching treatment as fixed factors. Significant relative fitness responses tested with Student t tests and LMM-corrected KR degrees of freedom are shown above each bar; post-hoc Tukey t tests with LMM-corrected KR degrees of freedom are shown among generations: * p < 0.05; ** p < 0.01; *** p < 0.001. Data deposited in the Dryad repository: http://dx.doi.org/10.5061/dryad.56bb4 [59]. (TIF) [file pbio.1002388.s004.TIF]

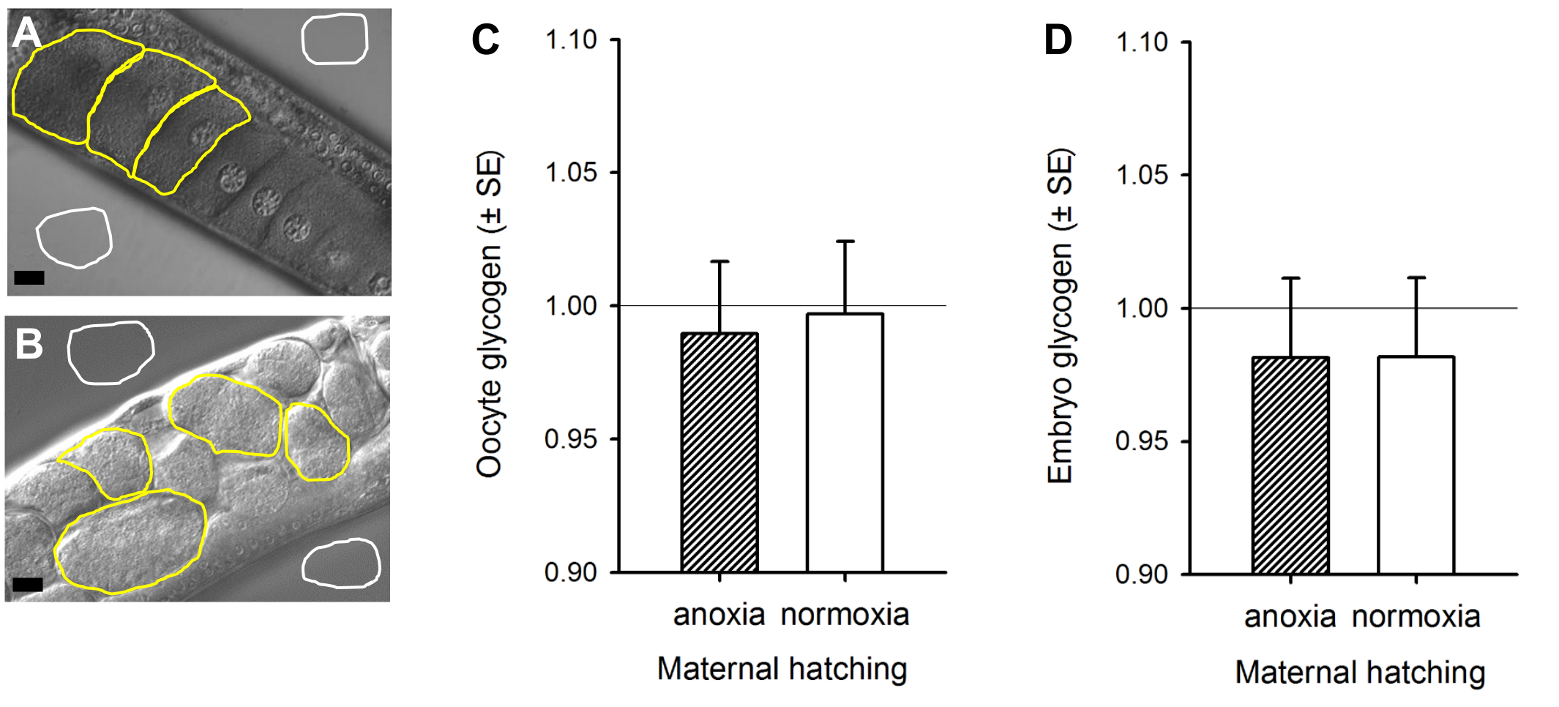

Supplement: S5 Fig — (A, B) Glycogen content is quantified in iodine-stained hermaphrodites at the time of usual reproduction during experimental evolution, following [50,52]. Illustrative photographs of stained hermaphrodites with oocytes (A) and unstained hermaphrodites with in utero embryos (B) are shown from ancestral hermaphrodites. The width of the black scale bar is 10 μm. The ratio of the mean pixel intensity of all the delineated oocytes or embryos (yellow lines) and the mean pixel intensity over the agar pad (white lines) was used for analysis (C and D, respectively). Data deposited in the Dryad repository: http://dx.doi.org/10.5061/dryad.56bb4 [59]. (TIF) [file pbio.1002388.s005.tif]

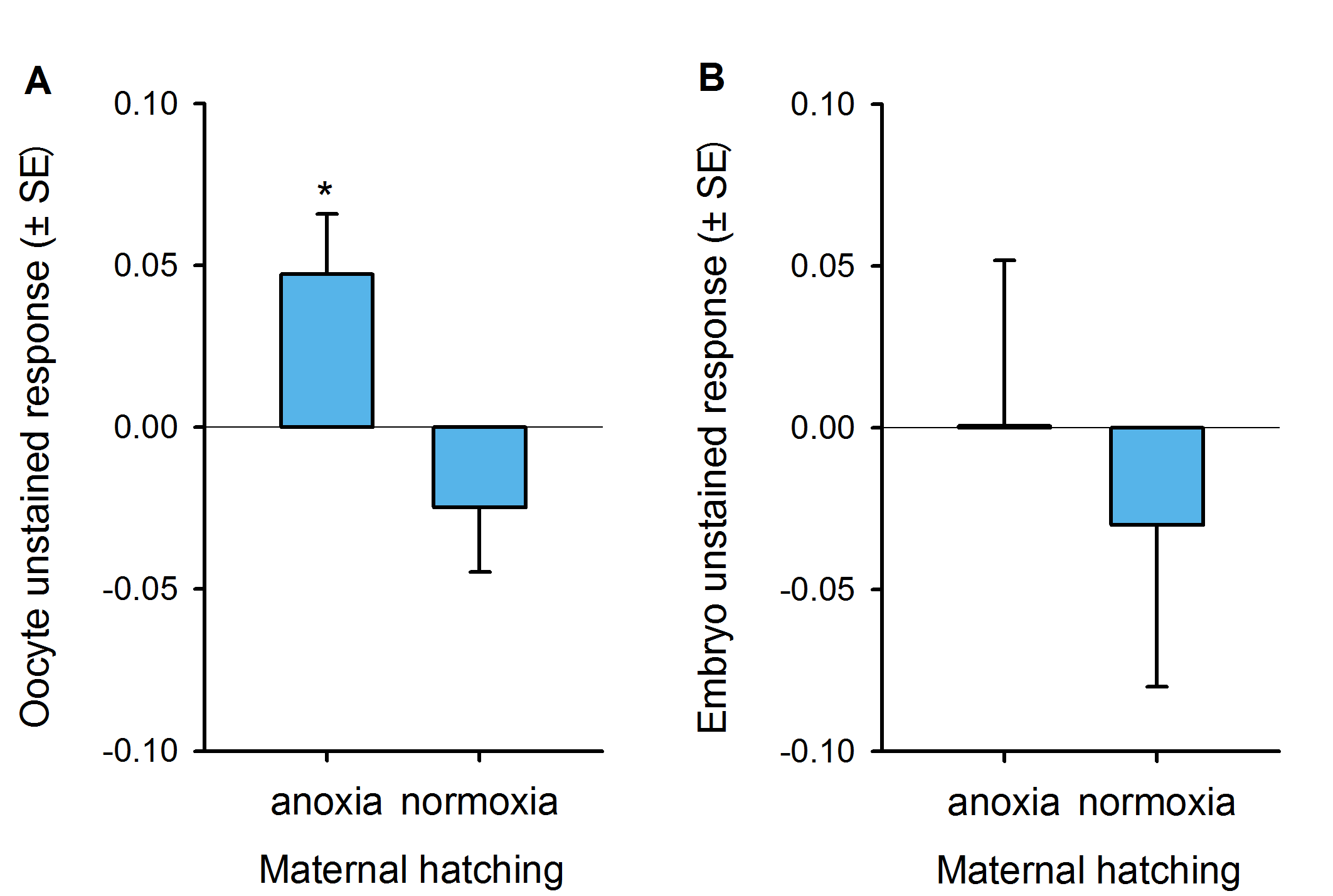

Supplement: S6 Fig — Oocyte (A) and in utero embryo (B) unstained hermaphrodite response of predictable populations at generation 60, relative the ancestor population (zero line). Except for iodine-staining, all other assay details were the same as those presented in Figs 6 and S5. Mean and error least square estimates are shown after LMM, taking replicate population and individual hermaphrodite as random factors and maternal hatching treatment as a fixed factor. Significant evolutionary response (Student t test, with LMM-corrected KR degrees of freedom) is shown above one of the bars: * p < 0.05. Data deposited in the Dryad repository: http://dx.doi.org/10.5061/dryad.56bb4 [59]. (TIF) [file pbio.1002388.s006.TIF]

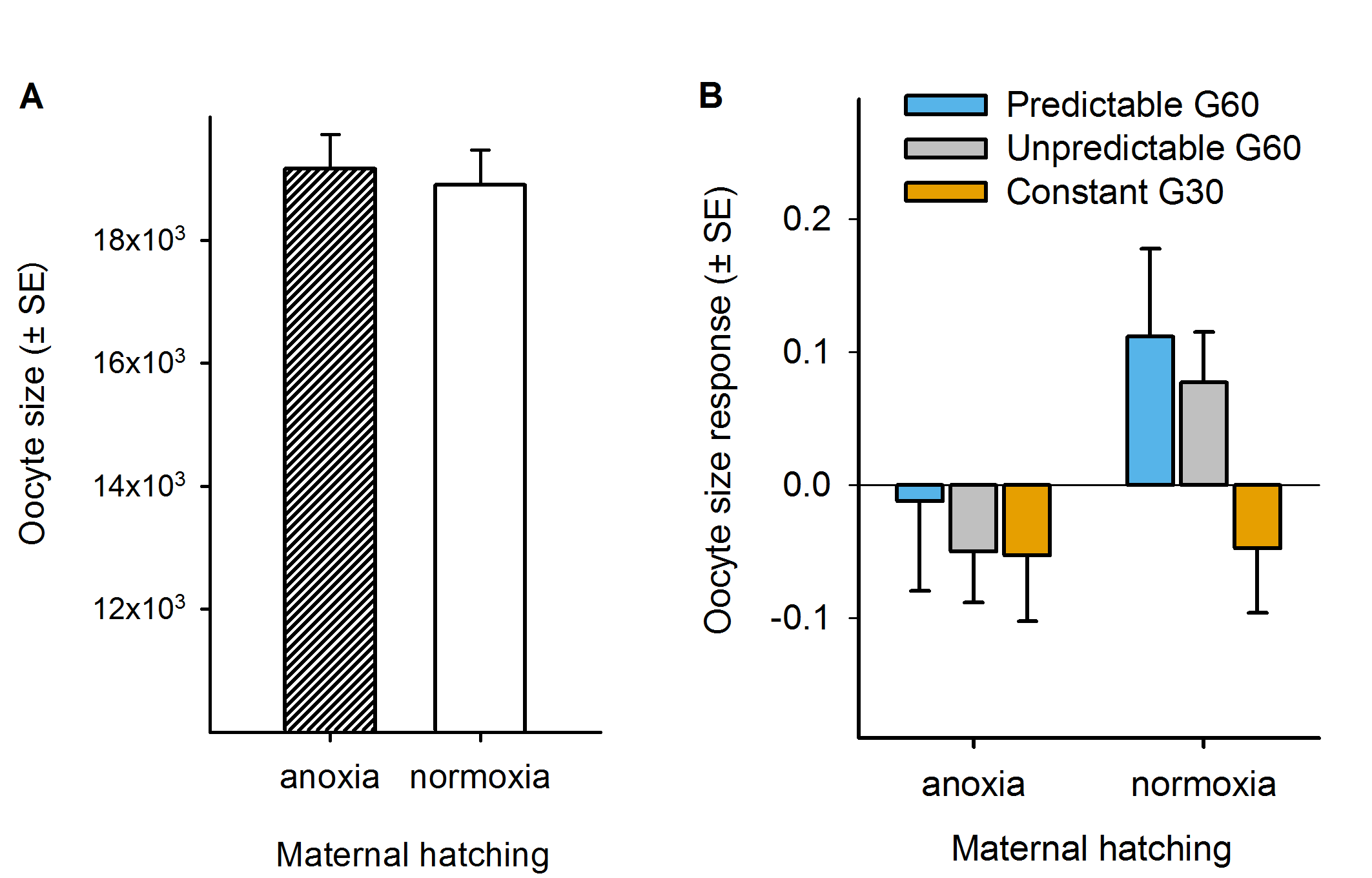

Supplement: S7 Fig — From the glycogen content assay, three oocytes were measured as the perimeter in pixels within hermaphrodites (white lines in S5A Fig). Shown are the absolute measurements of the ancestral population (A) and the oocyte size response in populations from all experimental regimes (B). Mean and error least square estimates are shown after separate LMM for each regime, taking replicate population and individual hermaphrodite as random factors and maternal hatching treatment as a fixed factor. There were no evolutionary responses, except perhaps in the unpredictable populations under maternal normoxia hatching (Student t test with LMM-corrected KR degrees of freedom: t8.6 p = 0.08). Data deposited in the Dryad repository: http://dx.doi.org/10.5061/dryad.56bb4 [59]. (TIF) [file pbio.1002388.s007.TIF]

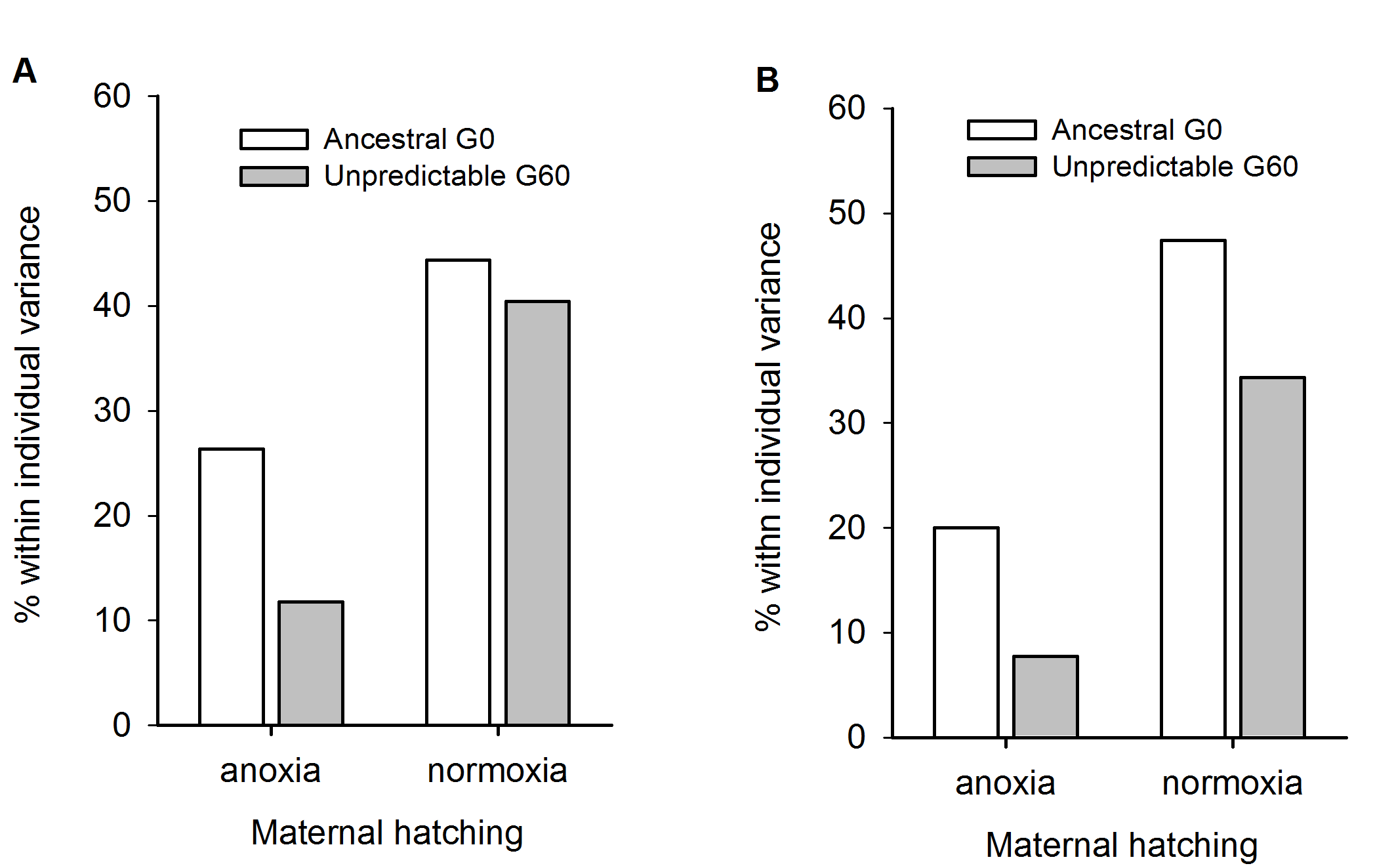

Supplement: S8 Fig — Percent of total variation explained by variation in oocyte glycogen content (A) or in utero embryo glycogen content (B). Separate LMMs were done per maternal hatching environment and regime. In the ancestral population, individual oocytes or embryos were modelled as a random factor nested within a random glass slide. In the unpredictable regime, individual oocytes or embryos were modelled as a random factor nested within a random glass slide nested within a random replicate population. Data had to be ln-transformed so that the models were identifiable and the algorithm reached convergence. Given unequal sample sizes per slide and, by design, different numbers of populations in each regime, results should be interpreted with caution, though there is no trend for an increase in brood trait variation, as would be expected with the evolution of a randomizing maternal effect. Data deposited in the Dryad repository: http://dx.doi.org/10.5061/dryad.56bb4 [59]. (TIF) [file pbio.1002388.s008.TIF]
